# Use of InSAR data for measuring land subsidence induced by groundwater withdrawal and climate change in Ardabil Plain, Iran

**DOI:** 10.1038/s41598-022-17438-y

**Published:** 2022-08-17

**Authors:** Zahra Ghorbani, Ali Khosravi, Yasser Maghsoudi, Farid Fazel Mojtahedi, Eslam Javadnia, Ali Nazari

**Affiliations:** 1 Faculty of Geodesy and Geomatics Engineering, K. N. Toosi University of Technology, Tehran, Iran; 2 Department of Civil and Environmental Engineering, Auburn University, Auburn, AL USA; 3 COMET, School of Earth and Environment, University of Leeds, Leeds, UK; 4 Sharif University of Technology, MSc graduate, Azadi Ave, Tehran, Iran; 5 Department of Surveying Engineering, Faculty of Engineering, University of Zanjan, Zanjan, Iran; 6 Department of Civil Engineering, KU Leuven, Leuven, Belgium

**Keywords:** Civil engineering, Hydrology

## Abstract

The Ardabil plain, with an approximate area of 1097.2 km^2^ in northwestern Iran, has experienced land subsidence due to intensive groundwater withdrawal and long seasons of drought in recent years. Different techniques have been used to investigate and evaluate subsidence in this region including: Global Positioning Systems (GPS), Levelling, and Geotechnical methods. These methods are typically expensive, time-consuming, and identify only a small fraction of the areas prone to subsidence. This study employs an Interferometric Synthetic Aperture Radar (InSAR) technique to measure the long-term subsidence of the plain. An open-source SAR interferometry time series analysis package, LiCSBAS, that integrates with the automated Sentinel-1 InSAR processor (COMET-LiCSAR) is used to analyze Sentinel-1 satellite images from October 2014 to January 2021. Processing of Sentinel-1 images shows that the Ardabil plain has been facing rapid subsidence due to groundwater pumping and reduced rainfall, especially between May 2018 to January 2019. The maximum subsidence rate was 45 mm/yr, measured at the southeastern part of the plain. While providing significant advantages (less processing time and disk space) over other InSAR processing packages, implementation of the LiCSBAS processing package and its accuracy for land subsidence measurements at different scales needs further evaluation. This study provides a procedure for evaluating its efficiency and accuracy for land subsidence measurements by comparing its measurements with the results of the GMTSAR and geotechnical numerical modeling. The results of geotechnical numerical modeling showed land subsidence with an average annual rate of 38 mm between 2006 and 2020, which was close to measurements using the InSAR technique. Comparison of the subsidence measurements of the Ardabil plain using the LiCSBAS package with results obtained from other techniques shows that LiCSBAS is able to accurately detect land deformation at large scales (~ km). However, they may not be optimized for more local deformations such as infrastructure monitoring.

## Introduction

Land subsidence caused by compaction of over-drafted aquifer systems and climate change has caused severe damage to agricultural lands, industrial activities, and urban infrastructure^1^. Land subsidence has been documented in several areas in the world including: Tehran, Iran^2^, Los Angeles^3^, Xi'an, China^4^, Calcutta^5^ and Las Vegas Valley and vicinity, Clark County, Nevada^6^. This phenomenon is a frequent environmental problem that needs to be examined in long-term, systematic research^7,8^. Therefore, a permanent monitoring system with a correct estimate of surface deformation is essential to show the existing threats posed by the land subsidence.

Over the past two decades, different techniques have been proposed and implemented to detect and evaluate land subsidence including Leveling^9,10^, Global Positioning Systems (GPS)^11,12^, and geotechnical techniques^13–15^. These methods are typically expensive, time-consuming, and provide continuous monitoring of land deformation over only a small fraction of the areas prone to subsidence. Compared with traditional measuring alternatives, the InSAR technique can detect a wider spatial range of surface deformation due to its broad spatial coverage and high accuracy^16,17^. The results obtained from this technique are capable of measuring small to large-scale displacements on the land surface. The most common families of time-series InSAR techniques used for land subsidence and land surface deformation measurements are multi-temporal interferometry methods including Persistent Scatterer–PS-InSAR (PSI)^18,19^ and Small Baseline Subset (SBAS)^20,21^. The PSI technique generates differential interferograms with one common master: identifying persistent point-wise reflectors, such as urban areas, with a high PS density^22^. The SBAS-InSAR technique, on the other hand, relies on a redundant network of image pairs with a short spatial and moderate temporal baseline. It detects the temporal evolution of surface deformations and increases the spatial coverage, especially over non-urban areas where the PS density may be low^21,22^. Compared to PSI, SBAS requires smaller Synthetic Aperture Radar (SAR) images and has a stronger ability to obtain nonlinear deformation information, particularly in regions with higher signal decorrelation^23^. Due to the advantages of SBAS over PSI, particularly in agricultural and non-urban regions, the SBAS-InSAR technique was considered for this study.

The SBAS-InSAR technique with Sentinel-1 data has been widely used to estimate surface displacement^7,24–33^. For example, Haghshenas Haghighi and Motagh^30^ studied the land subsidence hazard in Iran by country-scale analysis of sentinel-1 InSAR data through the years of 2014 to 2017. In their study, the subsidence zones were observed to be highly correlated with agricultural areas. A comparison of the land subsidence measurements and changes in groundwater level indicated that the subsidence in such areas are directly related to changes in groundwater level. Similar conclusions were made by^27,33^ when using Sentinel-1 SAR and SBAS for land subsidence monitoring in Hanoi, Vietnam, and Gorgan Plain, Iran, respectively. The results of^27^ also indicated that InSAR-derived land deformation data on a monthly scale could be used for characterization of the hydraulic head and aquifer properties in confined aquifer systems, even those experiencing significant subsidence.

Although data availability has been greatly improved by Sentinel-1, there are still barriers to making full use of this almost universal coverage and the vast amount of data involved. Also, the use of open-source software tools to process Sentinel-1A (S1A) images such as Generic Mapping Tools SAR (GMTSAR: an open-source—GNU General Public License—InSAR processing system designed with Generic Mapping Tools—GMT)^34^, Sentinel Application Platform (SNAP) (http://step.esa.int/main), and time series analysis by Stanford Method for PS (StaMPS)^35^ is challenging. These concerns are particularly salient in cases where long time series and large stacks of Sentinel-1 scenes are to be analyzed, often requiring more disk space for processing^36,37^.

Recently, an open-source SAR interferometry time series analysis package, LiCSBAS, has been developed and integrated with the automated Sentinel-1 InSAR processor (COMET-LiCSAR)^38^. Because LiCSBAS utilizes products published directly on the COMET-LiCS web portal (https://comet.nerc.ac.uk/COMET-LiCS-portal/), it does not need to pre-process and produce interferograms, thus saving significant time series processing time and disk space. In the LiCSBAS processing scheme, interferograms with many unwrapping errors are automatically identified by loop closure. Also, reliable time series and velocities are derived with the aid of masking using several noise indices. Furthermore, relative displacements on a large scale (> 100 km) and locally (2 km) can be measured with an accuracy of < 1 cm/epoch and ~ 2 mm/yr in velocity^36^. Epoch is the 12-day repeat cycles of the S1 sensor images. While being very promising and useful for the land deformation measurements, the performance of the LiCSBAS package has never been fully evaluated. This is particularly in the local context and mainly because of its generated interferograms which are multilooked by factors of 5 in the range and 20 in the azimuth directions, geocoded onto a 100 m grid, and spatially filtered for the main purpose of large-scale deformation monitoring and tectonic strain mapping^36^.

This study aims at evaluating the performance of the LiCSBAS package in measuring the long-term subsidence caused by groundwater exploitation and climate change. The evaluation is performed for the Ardabil plain in northwest Iran, from 2014 to 2021 using Sentinel-1 SAR data. Over the last decade, the Ardabil plain, one of the most important plains agriculturally and industrially, has experienced significant subsidence due to groundwater level fluctuations and climate change^39^. In the past, InSAR data for monitoring subsidence in the Ardabil plain was very limited. With the advent of Sentinel-1 satellite in 2014, abundant and useful SAR data have been provided for the Ardabil plain, which have the potential to detect ground deformation in high spatial and temporal resolution. In this research, the LiCSBAS package is used to estimate spatially and temporally detailed deformation time series of the Ardabil plain from the LiCSAR products for the period of October 2014 to January 2021To evaluate consistency of the LiCSBAS package in measuring land subsidence at scales relevant to Ardabil plain, the results of the LiCSBAS are first compared with the results of GMTSAR. GMTSAR is a well-tested tool for the analysis of satellite images and has been used for different applications such as land subsidence estimation^27,40–42^, landslide estimation^43,44^, and earthquake and other deformation estimation^45,46^. The motivation for choosing GMTSAR in this paper was due to its high processing performance, C-shell scripting susceptibility, good potential for parallel implementation, and free access compared to other commercial SBAS processing systems (such as SARscape—https://l3harrisgeospatial.com and GAMMA^47^). Then, the validity of SBAS-InSAR technique results are discussed using the time-series of land deformation obtained from permanent GPS observations, groundwater changes, and a sophisticated geotechnical based numerical modeling technique. The findings of this research provide researchers and practitioners with an accurate technique for measuring land subsidence over large areas due to the spatial coverage of available InSAR data and LiCSBAS’s efficient processing strategy. This is a critical need for groundwater management in areas that are prone to subsidence and provides a way for efficacy assessment of different strategies to limit land subsidence.

## Description of study area and research methodology

### Description of study area

**Figure 1:**
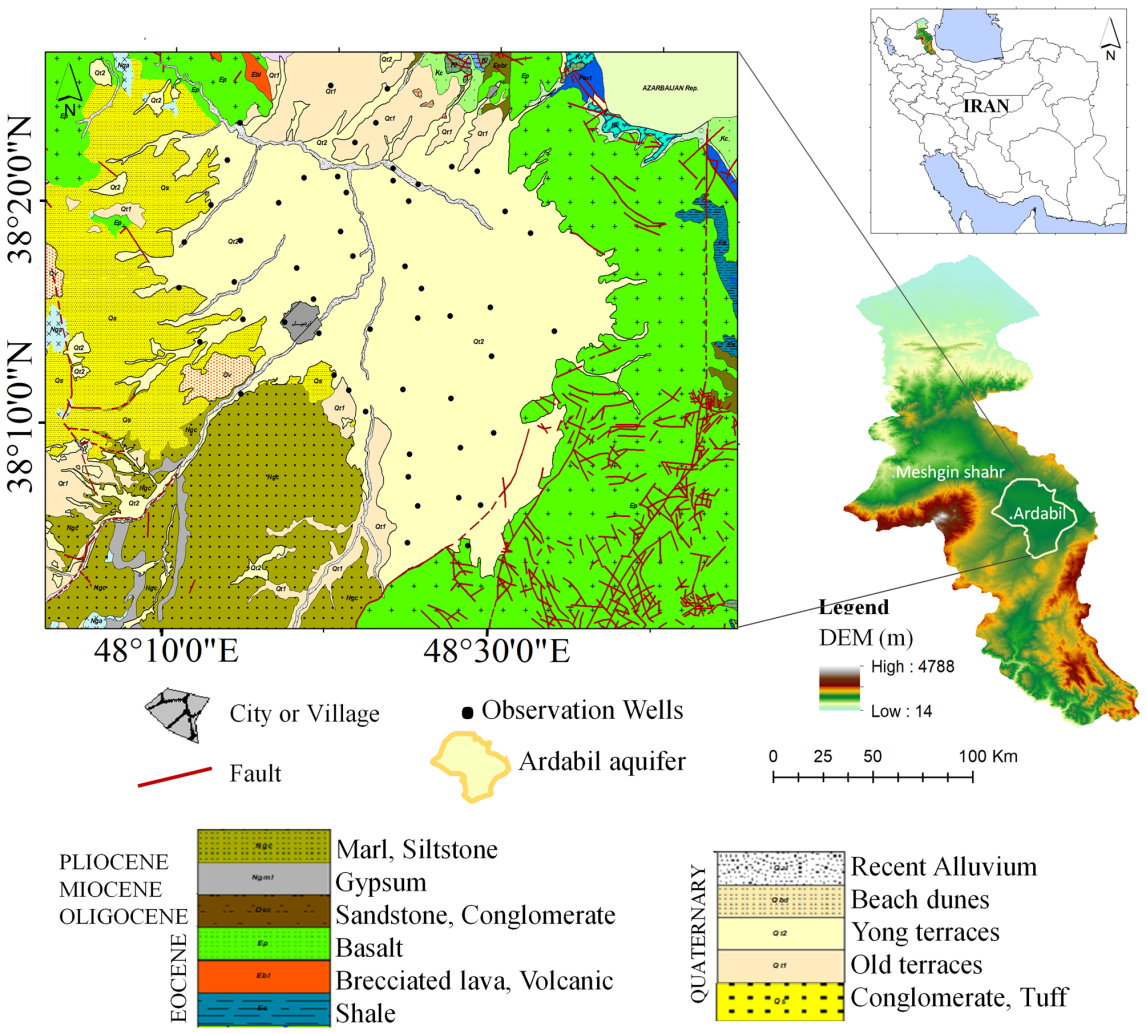
The geographical location of Ardabil plain. This figure was created using the QGIS version 3.14.0-Pi software (https://qgis.org/en/site/).

The Ardabil plain, with an approximate area of 1097.23 km^2^, is an inter-mountain plain located in northwestern Iran with geographical coordinates of 38°5' to 38°27' N and 48°9' to 48°37' E (Fig. 1).

The Ardabil plain has been formed out of Quaternary alluvial deposits due to weathering and erosion of surrounding mountains^48^. Conglomerate, volcanic ashes, and lahars are the predominant lithological outcrops in the west of the plain. There are abundant springs in these units which infiltrate into the groundwater and affect aquifer recharge**.** In the past, most springs, qanats, and rivers were used for water supply, but now water supply is mostly based on the exploitation of wells. Based on the results of geophysical studies, pumping test data, and drilling logs, Ardebil aquifer has a maximum thickness of 220 m and is mainly composed of gravel, sand, and a small amount of clay. The transmissivity of the aquifer varies between 50 and 2200 m^2^/day, and the specific yield ranges from 0.021 to 0.14. Storativity along with transmissivity and specific yield are key properties of the groundwater system. They are typically obtained in situ from pumping tests and reflect the response of aquifers and aquitards to groundwater head changes. Knowledge of their values is necessary to quantify available groundwater resources^49,50^.

**Figure 2:**
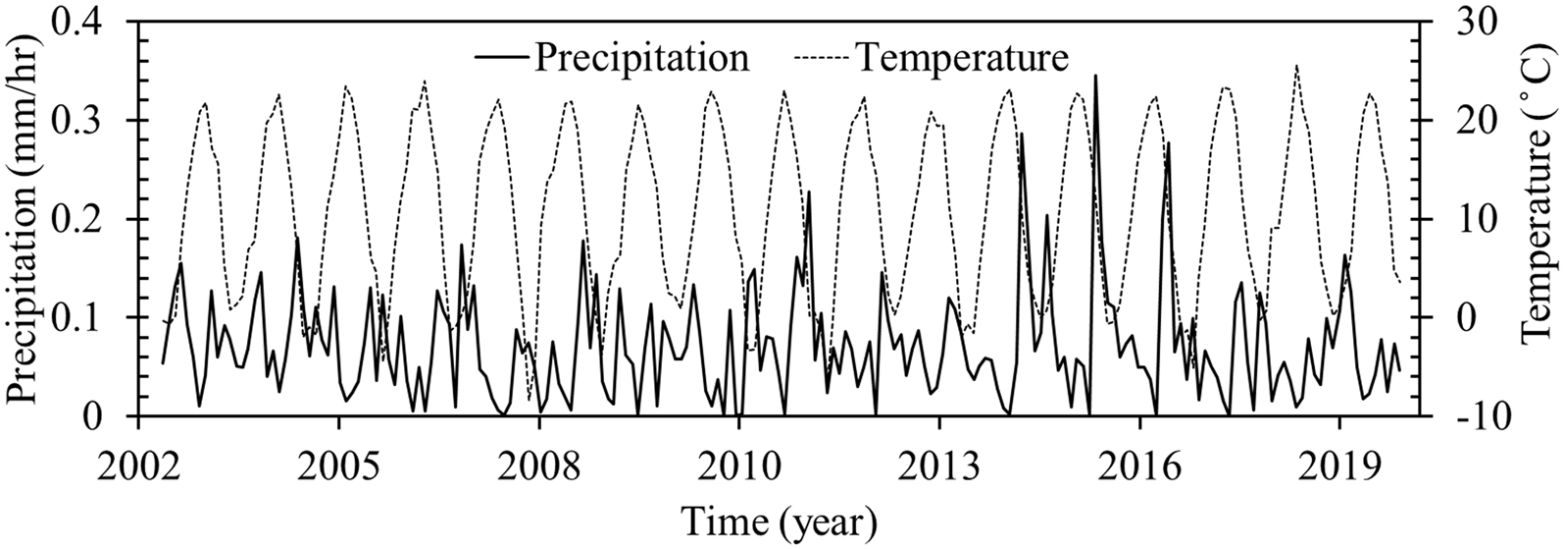
Total precipitation from (2000–2020) based on the TRMM rainfall estimate month data, and annual temperature rate from (2002–2020) based on the FLDAS datasets (https://das.gsfc.nasa.gov/fldas).

The general groundwater flows from other directions into the north–west of the plain^48^. More than 85% of exploited groundwater is used for agricultural activities, 14% is used for drinking purposes and 1% for industrial use. The yearly maximum and minimum groundwater levels are usually recorded in May and September, respectively^51^. Groundwater levels in the plain are directly affected by human activities and infrastructure, including industrial and agricultural activities^52^. The climate of the study area is cold and semi-arid. Figure 2 shows the precipitation and temperature times series of the region for the period of study. This data was obtained from Tropical Rainfall Measurement Mission (TRMM) satellite precipitation data (https://disc.gsfc.nasa.gov/datasets/TRMM_3B43_7) using the TRMM adjusted merged microwave infrared precipitation rate (in mm/hr) and Root-Mean-Square (RMS) precipitation-error estimates, and Famine Early Warning Systems Network Land Data Assimilation System (FLDAS) datasets (https://das.gsfc.nasa.gov/fldas). Times series of precipitation and temperature show average annual precipitation and temperature of ~ 113.8 mm/yr (mostly happening during the rainy season—August to April), and 11.1 °C (with the highest temperature in August and the lowest in February), respectively.

### Research methodology

#### InSAR data

**Figure 3:**
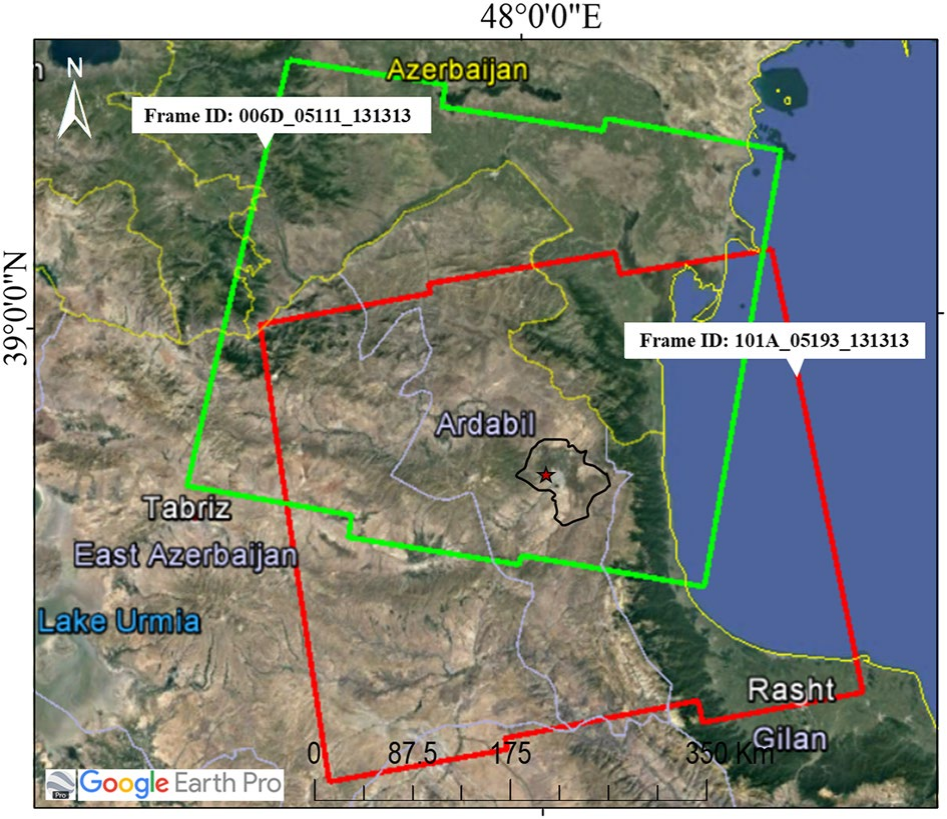
Geographical locations of SAR images used to study land subsidence in Ardabil plain (https://comet.nerc.ac.uk/COMET-LiCS-portal/). This figure was created using the Google Earth/Pro version 7.3.4.8573 software (https://earth.google.com/).

In this study, Sentinel-1 satellite images from October 2014 to January 2021 in a time interval of 12 days were used (Fig. 3). The Interferometric Wideswath (IW) mode was used, which creates images with a 250 km swath at 5 × 20 m spatial resolution. Details of the SAR images that were used for the InSAR time series analysis are presented in Table 1. We processed the LiCSAR generated interferograms over two LiCSAR frames covering Ardabil plain in ascending and descending orbits (LiCSAR frame ID 101A_05193_131313 and 006D_05111_131313) using LiCSBAS package. The interferograms were created with the GAMMA SAR software and filtered using an adaptive phase filter^38,47^. GeoTIFF files of unwrapped phase and coherence are published on the COMET-LiCS web portal on a consistent geographic frame basis in 250 km^2^. Both ascending and descending data were available for the whole or a specific period. The numbers of processed epochs for the ascending and descending frames were 137 and 136, respectively.

**Table 1:** Details of the SAR images used for the InSAR time-series analysis.

SAR Sensor	Orbit	(*λ*)	Revisit cycle	(*θ*°)	*N*	Temporal coverag	Mode
S1A-101	Ascending	C(5.56)	12 days	41.45	137	20141129–20200910	IW2
S1A-6	Descending	C(5.56)	12 days	40.22	136	20141006–20210114	IW1

*θ* Incidence angle of the sensor.

*N* Number of processed epochs.

#### LiCSBAS time-series analysis

**Figure 4:**
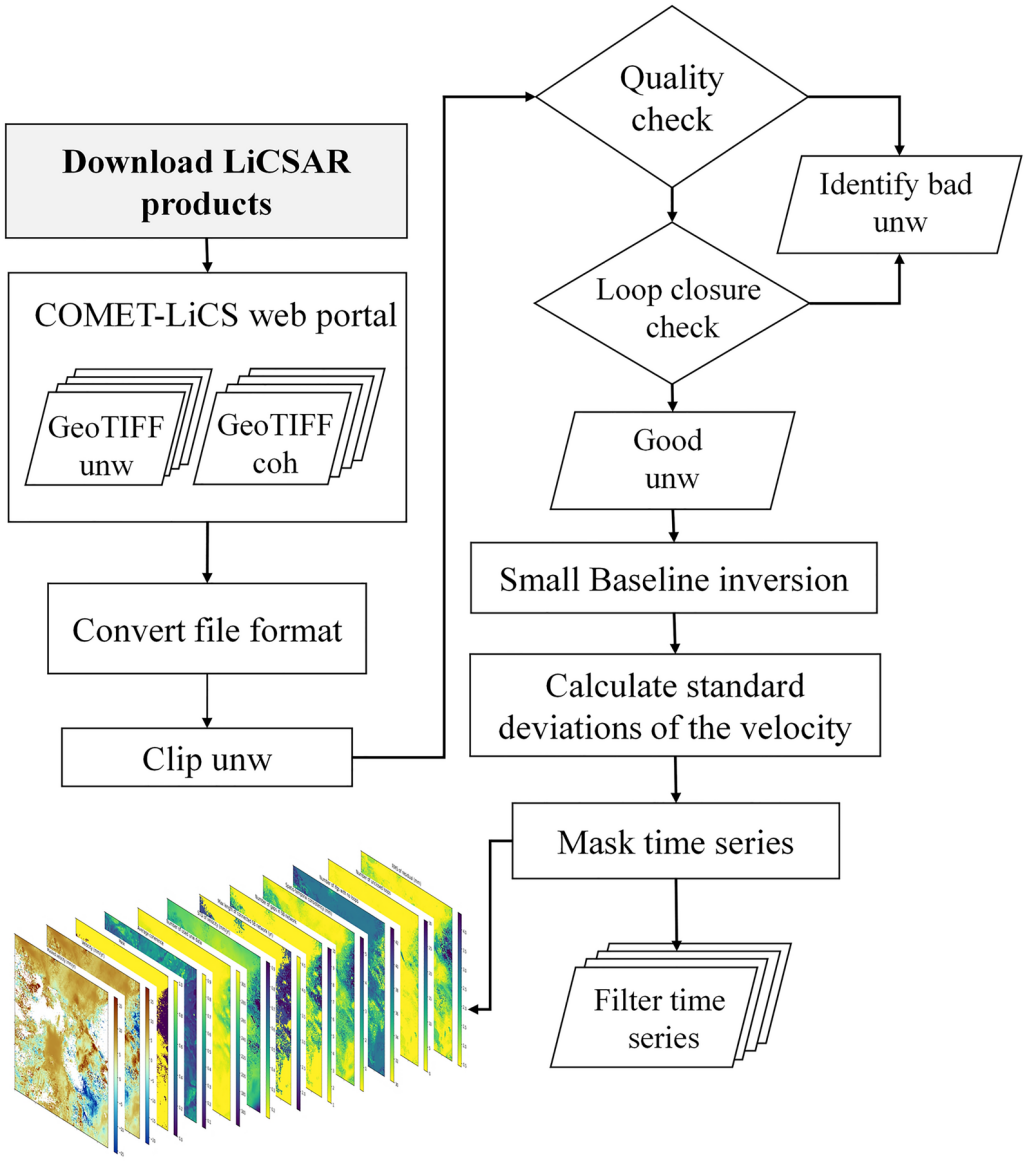
Workflow of LiCSBAS time-series analysis.

LiCSBAS is an open-source package in Python3 to carry out InSAR time series analysis using LiCSAR products (i.e. unwrapped interferograms and coherence) that are freely available on the COMET-LiCS web portal^36^. LiCSBAS can run all steps selected for analysis with specified parameters at once using a batch script. Figure 4 shows the workflow of LiCSBAS time-series analysis. The first step is to run the script, download LiCSAR products and then convert file format. In the next step, to reduce the impact of unwrapping errors in Ardabil plain, before the subsequent time-series analysis, pixels with a coherence of 0.1 are masked and then the data are unwrapped automatically using SNAPHU^53^. Thereafter, all the unwrapped data and corresponding coherence images are clipped to the Ardabil plain area. The coverage of unwrapped data and coherence threshold are set to < 0.5 and 0.06, respectively. The unwrapped interferograms are resampled and geocoded using the Shuttle Radar Topography Mission Digital Elevation Model (SRTM DEM 30 m). The COMET-LiCSAR system processes the InSAR data and generates interferograms that connect each epoch to three or four nearest acquisitions in time, backward and forward. LiCSBAS employs a modified small-baseline NSBAS approach^54,55^ for the time-series analysis. In the time-series analysis, deficient interferograms are identified and then discarded based on the coherence and coverage of the unwrapped data by checking loop closure^36,56^.

**Figure 5:**
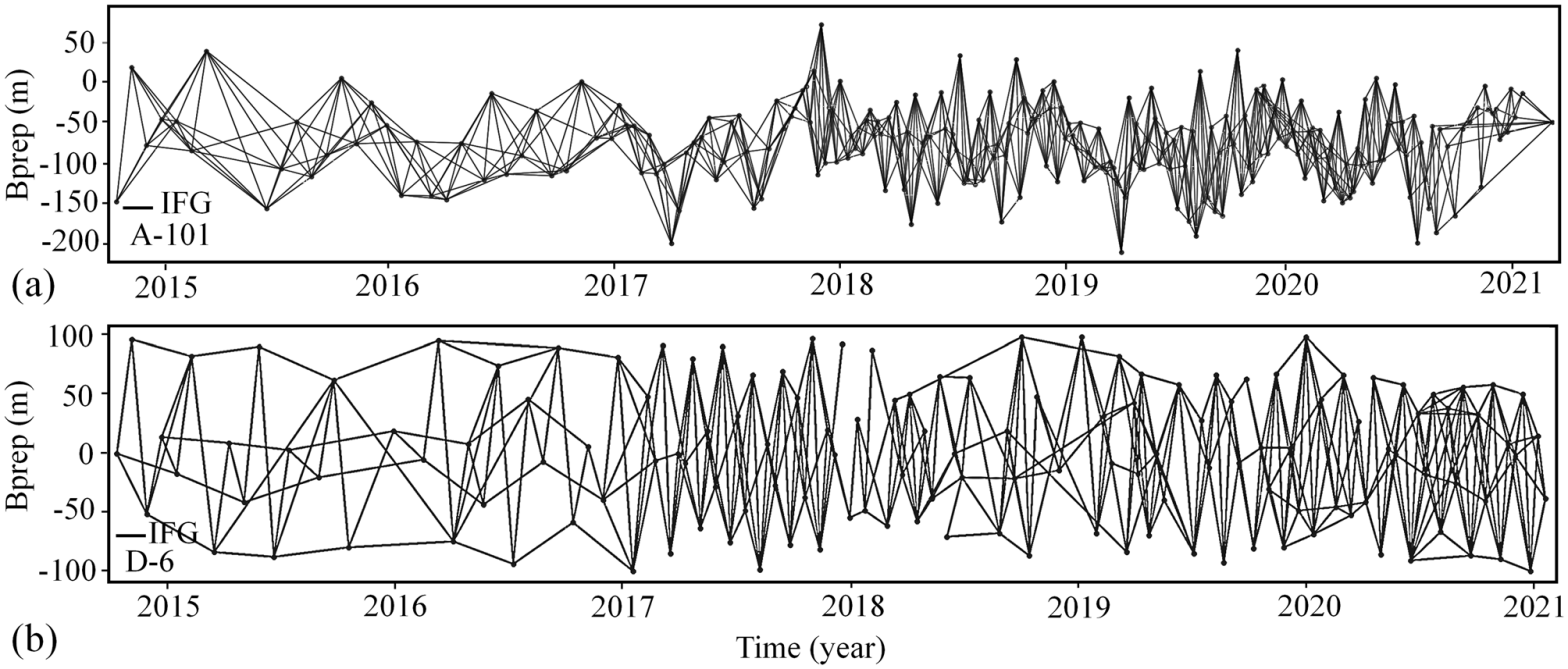
Perpendicular baseline configuration and network of the 453 and 365 interferograms formed from track (**a**) A-101 and (**b**) D-6 S1A images used in the study area.

The whole interferometric networks are then inverted for incremental displacements between the acquisition dates, using the least-squares method^36^, to estimate the displacement value of each pixel in the Ardabil plain. Overall, the baseline networks obtained from S1A images for the ascending (A-101) and descending (D-6) tracks include 453 and 365 interferograms, respectively (Fig. 5a and b). Interferograms that contain noisy data or do not pass a phase loop closure test (indicating severe unwrapping errors) are automatically excluded from the analysis^57,58^. Finally, the maps are high-pass filtered in time and low-pass filtered in space using a Gaussian filter kernel^59^, to separate noise components from the displacement time series (set to 0.50 year-182 days).

In order to compare the LiCSBAS and permanent GPS data, the following equation was used to obtain GPS*LOS* measurements in LOS direction^60^:

1 $${GPS}_{LOS}={GPS}_{up}cos\theta -\left({GPS}_{e}cos\alpha -{GPS}_{n}sin\alpha \right)sin\theta $$

where $\alpha$ is the azimuth of the LOS vector, *θ* is the incidence angle, and *GPS*_*up*_, *GPS*_*e*_ , *GPS*_*n*_ are the velocities in vertical directions, the eastwest and northsouth, respectively.

## Results

We estimated the surface displacements in the Ardabil plain using LiCSBAS. Then, the accuracy and consistency of the results were analyzed in both LiCSBAS and GMTSAR InSAR tools.

### Interpretation of the S1A data by LiCSBAS velocities

**Figure 6:**
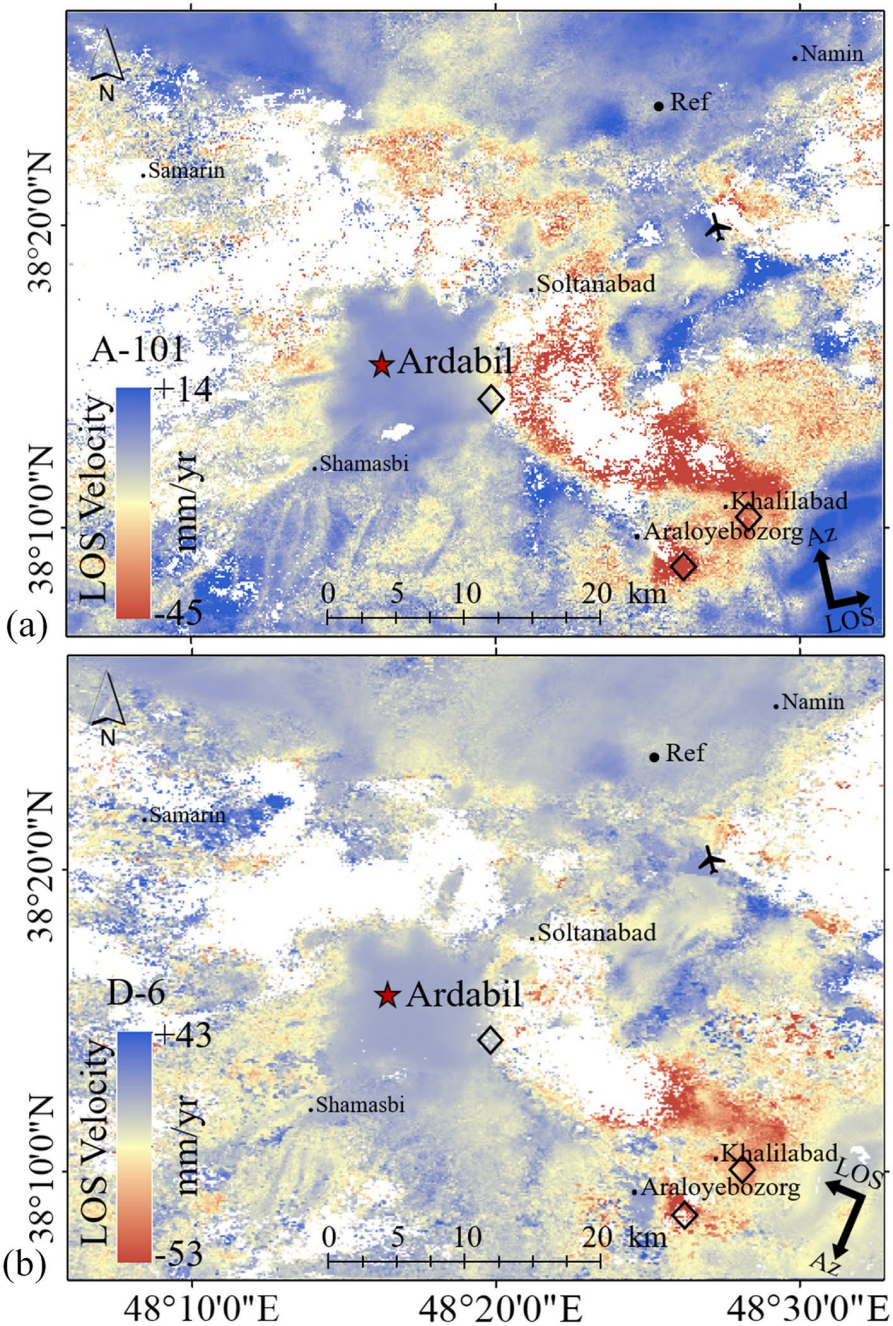
Mean LOS velocity of the results S1A images by LiCSBAS and displacement relative to the reference point. (**a**) From October 2014 to January 2021 for D-6 track, and (**b**) From November 2014 to September 2020 for A-101 track. This figure was created using the QGIS version 3.14.0-Pi software (https://qgis.org/en/site/).

The mean velocity maps derived from the time series analysis of S1A images by LiCSBAS are shown in Fig. 6. Several surface displacement signals including subsidence and uplift were detected in the Ardabil plain. Despite having almost all pixels masked in highly vegetated areas due to decorrelation, more than 110 thousand valid pixels (60%), mainly distributed on the Ardabil plain, still remain (Fig. 6). To obtain meaningful and accurate displacement measurements, a suitable reference area, where the displacement signal is almost zero and marked by the black circle in Fig. 6a and b, was considered for the study area. The calculated mean LOS velocities of S1A images from A-101 (measured in the period of November 2014 to September 2020) and D-6 (measured in the period of October 2014 to January 2021) tracks are between −45 mm/yr to + 14 mm/yr and between −53 mm/yr to + 43 mm/yr, respectively. Since the displacement is illustrated in LOS direction, positive and negative velocities represent the motion of the ground towards and away from the satellite, respectively. The mean LOS velocity values show a maximum displacement rate of 45 mm/yr in the LOS direction in an area located in the southeast part of the plain. This area is categorized as a cropland area with excessive groundwater extraction. Results presented in Fig. 6 also show signs of land subsidence in the cropland areas in the northwest region with a maximum displacement rate of 20 mm/yr. Other areas of the plain show no sign of significant subsidence. In Samarin, especially on its side towards Sabalan peak, there is also a large motion of + 43 mm/yr towards the satellite as presented in Fig. 6b. This area is in the mountainous part of the plain and has the lowest alluvial thickness (10–30 m). It is therefore expected to see the lowest rates of land displacement in this area, which is different from our observations in the area. Different factors can cause the observation of such large motion toward the satellite: (1) The measurement of InSAR observations is relative and highly dependent on the choice of the reference area considered for analysis. This reference area is assumed to be a stable region with no displacement. However, in practice, if the reference area is subjected to subsidence, this may cause observation of an uplift in regions with no displacement; (2) The study area is located in a mountainous area, 18 km from Sabalan mountain. In mountainous areas, topography-related tropospheric effects can be significant. This could result in an uplift signal in that region; (3) Although we try to remove the unwrapping errors in LiCSBAS, some unwrapping errors might still exist and cause some unreal signals such as the uplift signal; (4) It can be the effect of phase bias due to existence of short-interval interferograms. The size of the cumulative loop closure phases can be significantly reduced as the length of the short-interval interferograms in the loop increases^61,62^. This can lead to the observation of an additional uplift in the area.

**Figure 7:**
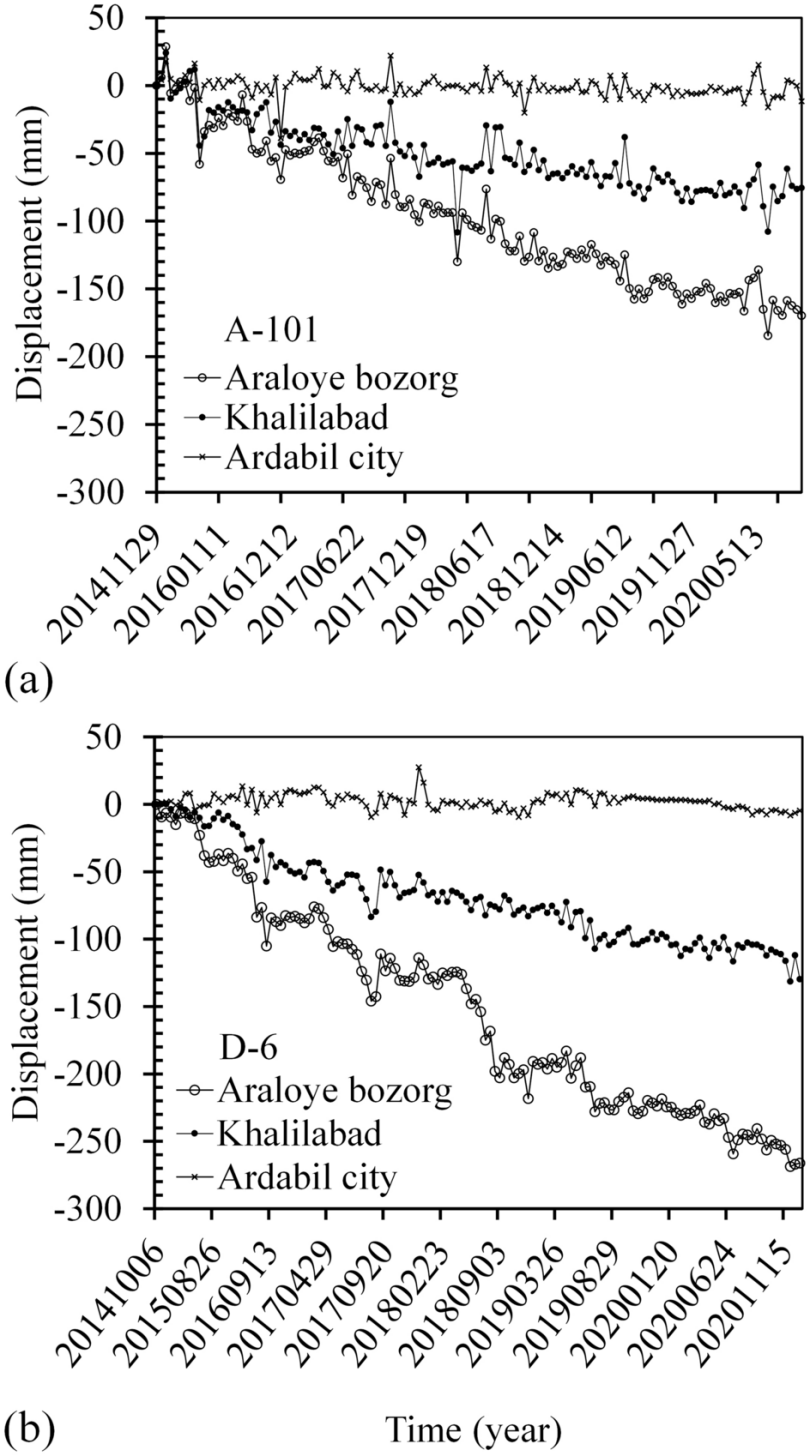
Displacement time-series from S1A images at three representative locations. (**a**) From October 2014 to January 2021 for D-6 track and, (**b**) From November 2014 to September 2020 for A-101 track.

Figure 7 shows displacement time-series in three different sites (diamonds in the Fig. 6), near the cities of Ardabil, Khalilabad, and Araloyebozorg. Khalilabad and Araloyebozorg are both located in the southeast with the highest rates of subsidence, and obtained using LiCSBAS. Results indicated a decreasing trend of displacement with time over the years of 2014 to 2021, with a maximum LOS displacement rate of 45 mm/yr near Araloyebozorg. Also, results indicate an increase in the subsidence rate in 2018. Different factors are responsible for this high rate of displacement in the southeast.

**Figure 8:**
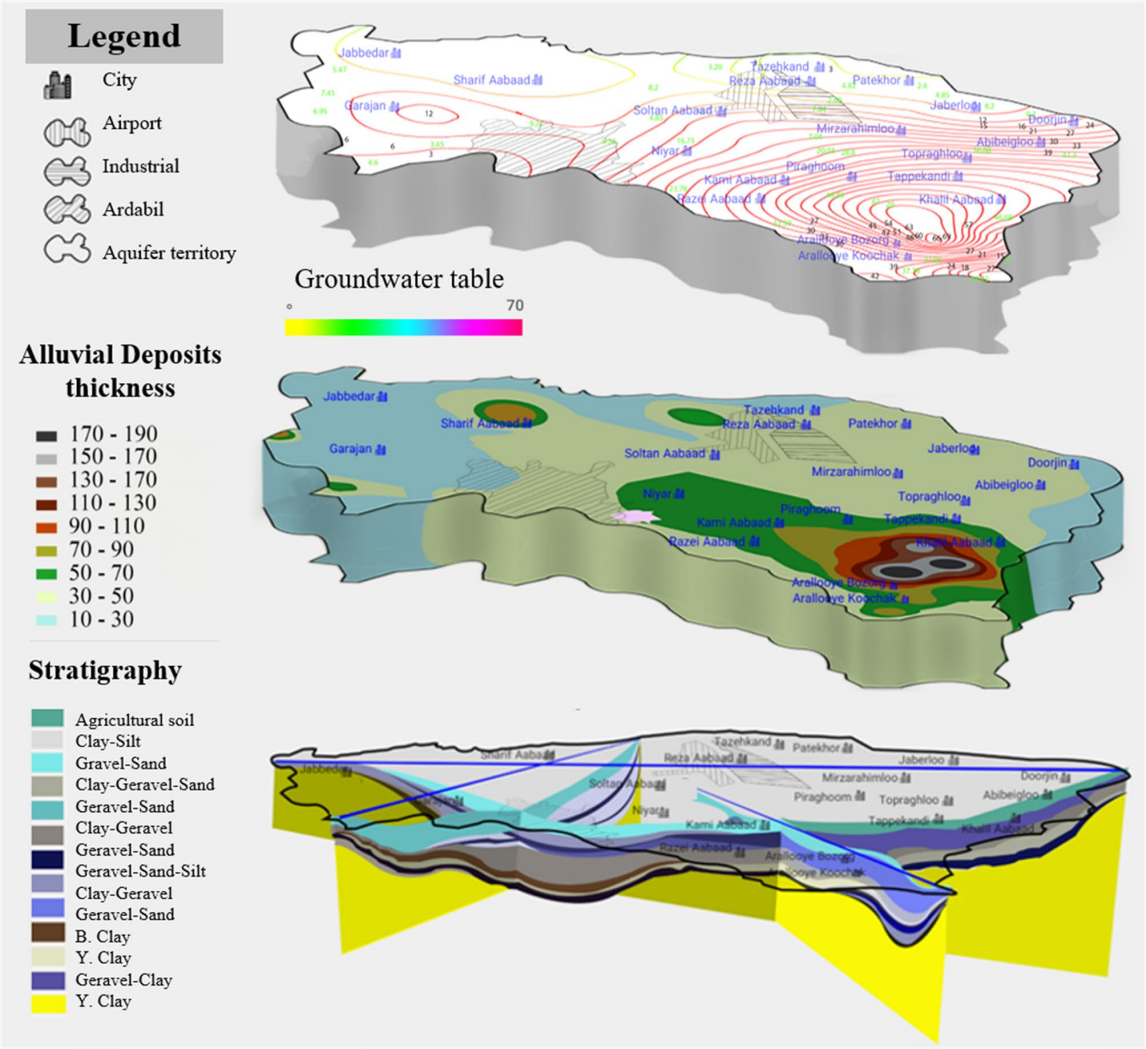
Parameters involved in the issue of subsidence: groundwater table, alluvial deposits thickness, type of soil layers and stratigraphy.

Figure 8 shows some of the factors that play the most significant roles in land subsidence, including groundwater table, alluvial deposit thickness, type of soil layers and stratigraphy. According to Fig. 8, the southeast part of the plain has the maximum alluvial thickness of 190 m, especially in areas near the cities of Araloyebozorg and Khalilabad. According to studies by Guzy and Malinowska^48^, this area has a clay layer with a thickness of more than 120 m, starting from a depth of about 25 m. The thickness of the sand layer is only 25 m in this area. Also, the ratio of fine-grained to coarse-grained materials in this area is higher compared to the other parts of the plain, increasing potential for land subsidence^63,64^. The groundwater table measurements from the observation wells indicate an average depth of 60–70 m in the southeast, which are deepest in the whole plain. The central part of the plain has the highest illegal and legal well density with significant water withdrawal, and therefore, it is expected to observe higher rates of subsidence in this area compared to other parts of the plain. However, since the groundwater level in this area is high (due to the bowl-shaped nature of the plain), the plain in this area experiences lower rates of subsidence with groundwater withdrawal.

### Consistency assessment

**Figure 9:**
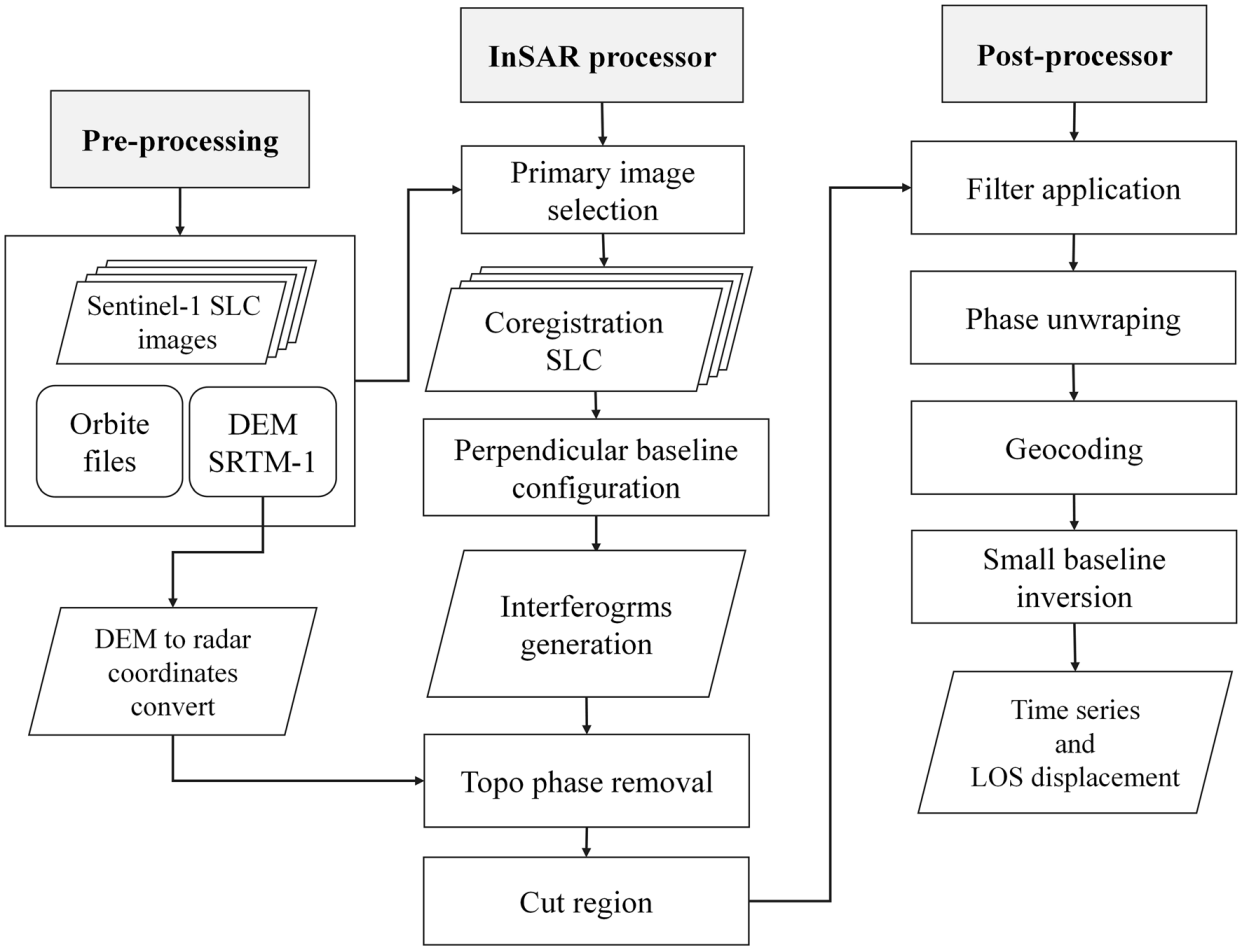
Flowchart of the SBAS-InSAR technique by GMTSAR used in this study.

To evaluate consistency of the LiCSBAS package in measuring land subsidence of the Ardabil plain, results of the package were compared with subsidence measurements obtained using the GMTSAR^63^. For this purpose, S1A images of 30 (A-101) and 28 (D-6) tracks, spanning March 2017 to January 2019, were analyzed using the GMTSAR. The interferometric process by GMTSAR based on SBAS-InSAR followed three main steps: First, a preprocessor was used for each satellite data type to convert the native format and orbital information into a generic format. Second, the InSAR processor was used to focus and align stacks of images and convert map topography into phase which is then used in radar coordinates, and form the complex interferogram. The topographic phase component was corrected using the SRTM 30 m DEM. Third, a postprocessor was applied based on GMT, to filter the interferograms and construct interferometric products of phase, coherence, phase gradient, and LOS displacement in both radar and geographic coordinates^65^. Figure 9 shows the workflow of time-series analysis by GMTSAR. GMTSAR uses an adaptive power spectrum filter and a Gaussian filter for the unwrapping process (set to default filter parameters of alpha = 0.1 and patch size = 32). In general, the numbers of interferograms associated with a perpendicular baseline of smaller than 120 m and a temporal baseline of smaller than 130 days obtained from S1A images of (A-101) and (D-6) tracks are 101 and 97, respectively. Using the least-squares method, the displacement value of each pixel was estimated in the Ardabil plain. To make the results comparable with the LiCSBAS/LiCSAR processing chain, the same multilooking factors of LiCSAR data were employed for GMTSAR processing and the coherence threshold was set to 0.06.

**Figure 10:**
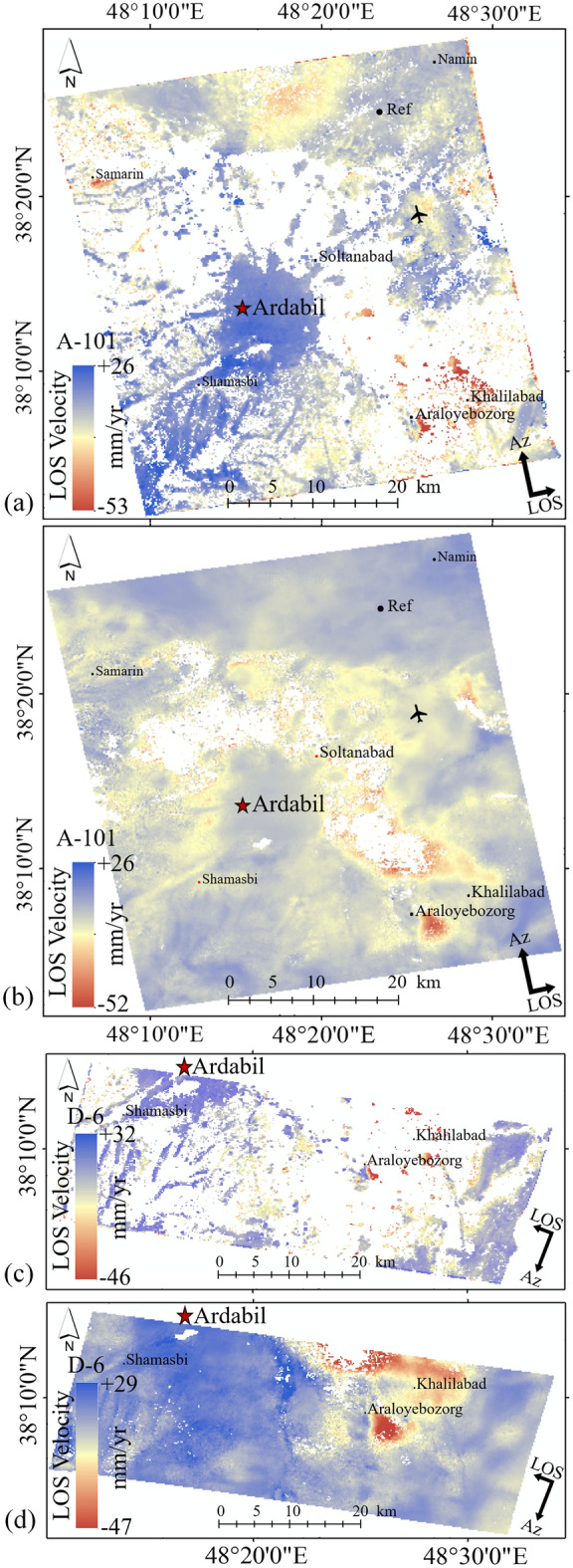
Comparison of the LOS displacement maps from S1A images (March 2017 to January 2019) for A-101 track using (**a**) GMTSAR, (**b**) LiCSBAS, and for D-6 track using (**c**) GMTSAR and (**d**) LiCSBAS. This figure was created using the QGIS version 3.14.0-Pi software (https://qgis.org/en/site/).

Figure 10 compares the LiCSBAS velocities with those measured using GMTSAR for the period of March 2017 to January 2019 for A-101 track. Figure 10a shows the maximum subsidence rates measured using the GMTSAR and Fig. 10b shows the results obtained using LiCSBAS. As observed in this figure, in terms of the maximum subsidence rates, both packages show close values of −53 mm/yr (GMTSAR) and −52 mm/yr (LiCSBAS). For the D-6 track, these values changed to −46 mm/yr (using GMTSAR) and −49 mm/yr (using LiCSBAS), as observed in Fig. 10c and d. Both GMTSAR and LiCSBAS results showed a major land subsidence concentrated in the southeast of the Ardabil plain, near the city of Araloyebozorg and Khalilabad. However, the difference between LiCSBAS and GMTSAR results is the number of masked pixels in areas of low coherence which is significantly larger in GMTSAR.

**Figure 11:**
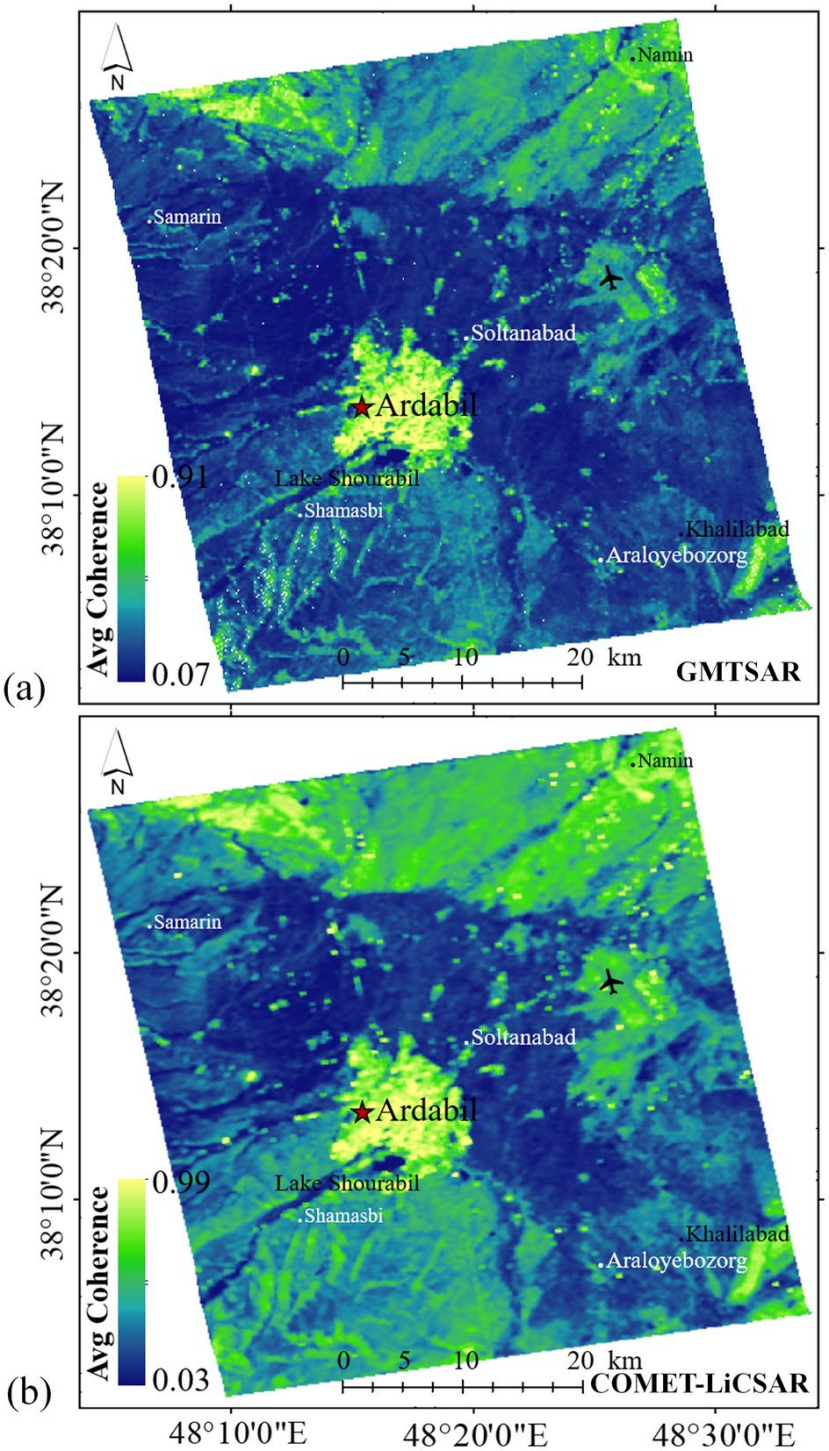
Comparison of the average coherences for the study area: (**a**) GMTSAR and (**b**) COMET-LiCSAR system. This figure was created using the QGIS version 3.14.0-Pi software (https://qgis.org/en/site/).

In Fig. 11, the average coherence maps (March 2017 to January 2019) in Ardabil plain using GMTSAR and COMET-LiCSAR system are presented. Based on the results presented in Fig. 11a and b, the values of average coherence for GMTSAR and COMET-LiCSAR are 0.27 and 0.37, respectively. Both results showed high coherence values (higher than 0.7) in areas around the city and part of the airport. Low coherence values (less than 0.3) were observed in areas such as croplands on the outskirts of Ardabil plain, vegetated sides of the Talesh heights in the eastern side of Ardabil plain, Shourabil Lake located in the south of Ardabil city, steep slope, rangelands, and vegetated slopes of Sabalan mountain in the western part of Ardabil plain.

**Figure 12:**
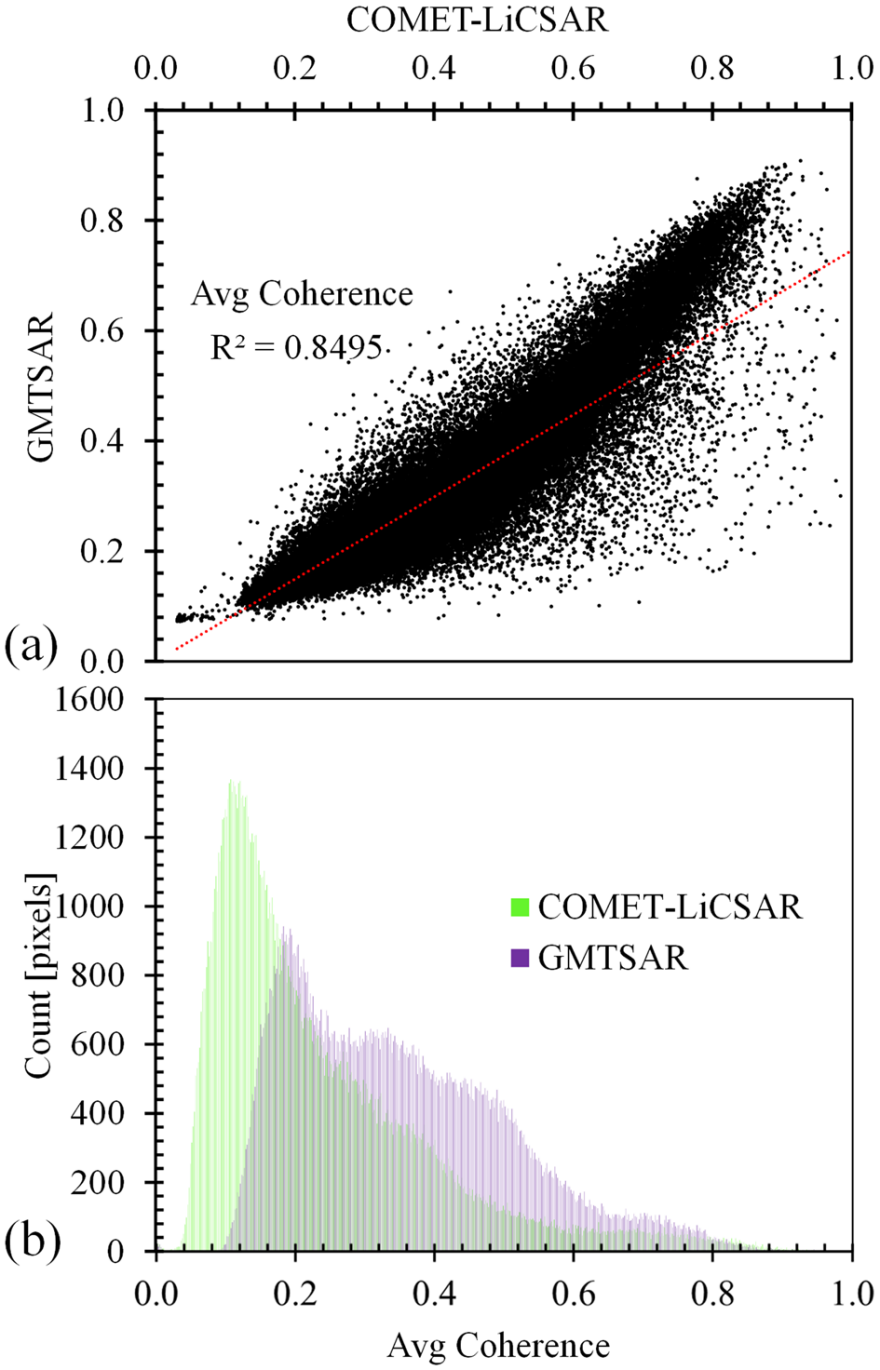
Comparison of the average coherence in GMTSAR and COMET-LiCSAR system using (**a**) Scatter plots and (**b**) Histograms.

Figure 12 shows scatter plots (Fig. 12a) and histograms (Fig. 12b) of average coherence obtained using GMTSAR and COMET-LiCSAR system. As presented in Fig. 12a for the coherences of 0.15–0.7, the pixels are distributed along the red line, showing consistency between the GMTSAR and COMET-LiCSAR systems. This figure clearly shows a good correlation with an *R*^*2*^ value of 0.84. Based on the results presented in Fig. 12b, the mean coherence difference between GMTSAR and COMET-LiCSAR is 0.1, with a standard deviation (Std) of 0.01. It is observed that the coherence values produced by GMTSAR are slightly lower than the COMET-LiCSAR system.

**Figure 13:**
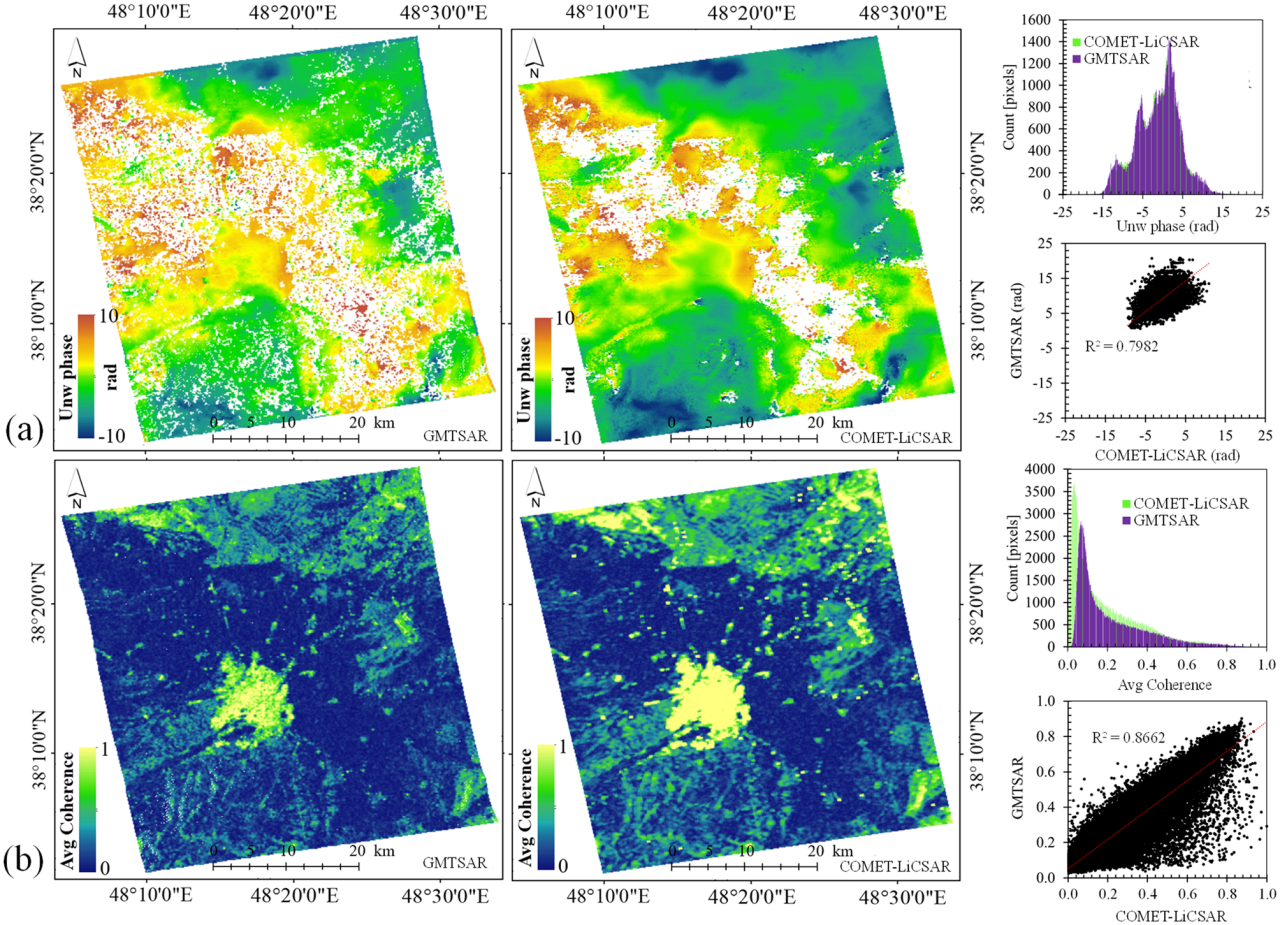
Comparison of the unwrapped interferograms and the coherence values used in GMTSAR and COMET-LiCSAR system: (**a**) The unwrapped phase pair 20170423–20170610. (**b**) The coherence pair 20170423–20170610. This figure was created using the QGIS version 3.14.0-Pi software (https://qgis.org/en/site/).

Also, for further comparison of differences in coherence values of the two systems (GMTSAR and COMET-LiCSAR), a pair of unwrapped interferogram phases (20170423-20170610) under challenging conditions were analyzed (Fig. 13). As presented in Fig. 13a and b, despite its substantially higher coherence values, the COMET-LiCSAR unwrapped interferogram phases for the scene pair are not different from GMTSAR results for most parts of the Ardabil plain (*R*^*2*^ = 0.79 and Std = 0.06), and the interferometric phase results have similar patterns. This behavior may be the result of using different approaches to calculate the unwrapped phase using the applied Gaussian filter.

The major difference is in the employed SBAS algorithm time series processing stage. In the implementation of the SBAS algorithm by GMTSAR, whenever there is one NaN value in any of the unwrapped phases, it will skip the whole column before doing the inversion and hence the pixel gets masked out. However, LiCBAS only removes that NaN value from the observation vector and will do the inversion using the remaining valid unwrapped phases. It should also be noted that even in the case of gaps in the small-baseline network (mainly due to decorrelation associated with factors such as vegetation or snow cover), LiCSBAS is able to do the inversion by imposing a linear temporal constraint across pixels with network gap. This explains the larger coverage of the LiCSBAS estimated velocity compared to the one obtained using GMTSAR. Particularly, in areas with relatively lower coherence, LiCSBAS seems to provide better estimate of the land subsidence.

**Figure 14:**
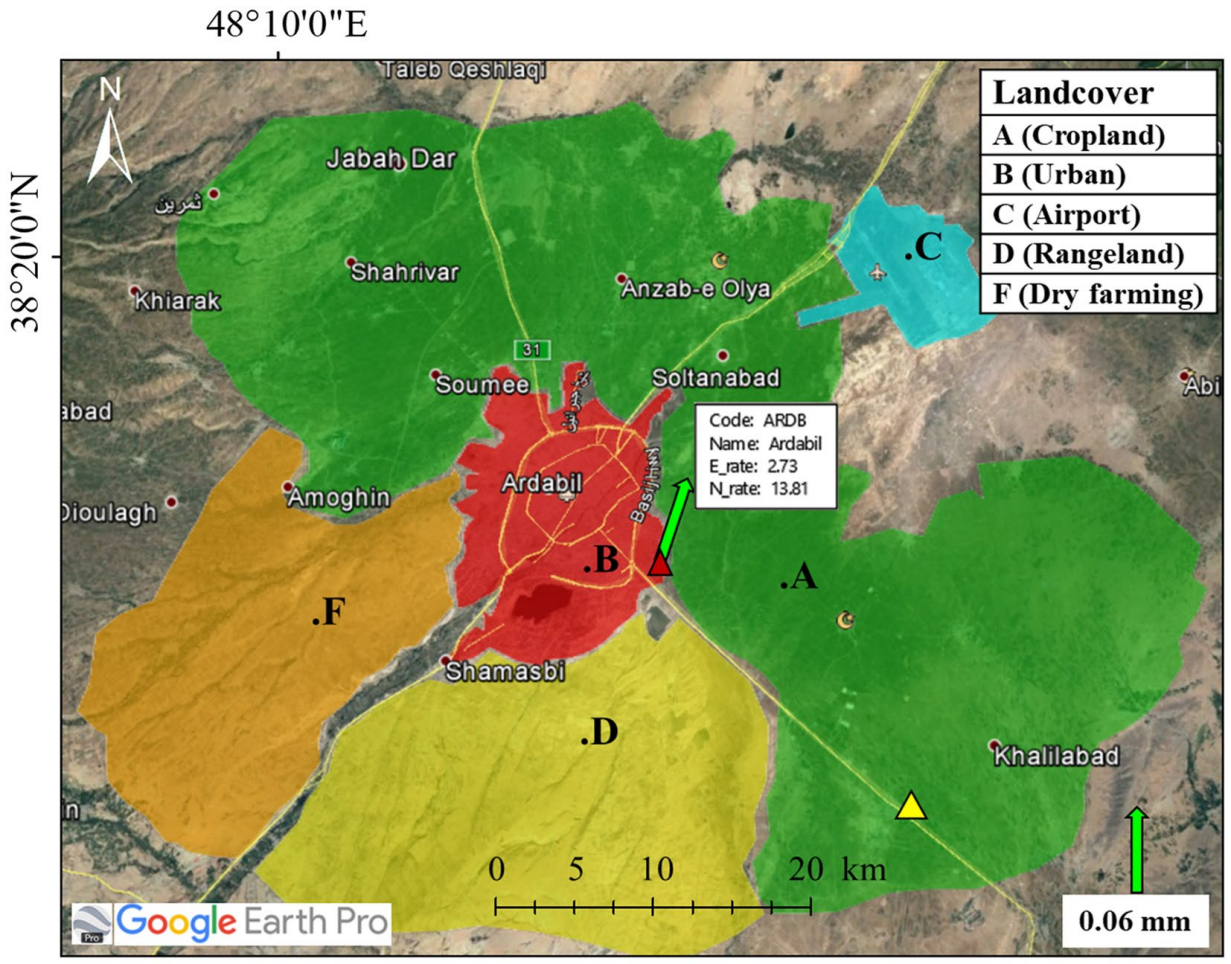
Land cover type and classification of the Ardebil plain. This figure was created using the Google Earth/Pro version 7.3.4.8573 software (https://earth.google.com/).

While LiCSBAS is able to accurately detect large-scale land deformation (~ km) of the plain, its accuracy may be considerably affected by the land cover and use type of the area under investigation. Land subsidence measurements of the Ardebil plain obtained using the LiCSBAS and GMTSAR packages were used to evaluate how land use type and cover affect land subsidence estimation using the two packages. For this evaluation, the plain was classified into five different classes based on their landcover types (Fig. 14): Class A is a cropland area that has the highest potential for land subsidence; Class B is the City of Ardebil, classified as urban area with the largest population and located at the central part of the plain; Class C is the airport and its runways, which are located very close to the area with the most subsidence; Classes D and F mainly consist of rangeland and dry farming respectively. The displacement rates of these class regions were then estimated using LiCSBAS and compared with those obtained using GMTSAR.

According to Table 2, the displacement velocities of LiCSBAS at cropland area for A-101 and D-6 track show subsidence at rates of 52 mm/yr and 47 mm/yr, respectively, which are consistent with GMTSAR. The Std of differences between the derived results in cropland area for both tracks are 0.09 mm/yr and 2.46 mm/yr, respectively. To evaluate consistency between the LiCSBAS and GMTSAR packages, a time series sample of subsidence behavior in Class A region was used.

**Table 2:** Information generated for displacement velocity maps due to subsidence at the landcover.

Frame (S1A)	A-101						D-6					
	LiCSBAS			GMTSAR			LiCSBAS			GMTSAR		
Area	Pixel	Vel_min Std (mm/yr)		Pixel	Vel_min Std (mm/yr)		Pixel	Vel_min Std(mm/yr)		Pixel	Vel_min Std (mm/yr)	
Plain	128,736	−52.73	6.67	99,003	−53.22	6.58	43,708	−47.51	6.64	20,942	−46.44	4.18
A (Cropland)	38,010	−51.83	7.65	9346	−51.19	8.16	10,107	−47.51	8.34	1195	−46.44	7.94
B (Urban)	6363	−8.43	2.21	6508	−7.91	2.29	1606	0.93	1.31	1440	−4.52	3.84
C (Airport)	2590	−23.43	2.73	2407	−18.73	3.55	NaN	NaN	NaN	NaN	NaN	NaN
D (Rangeland)	15,218	−14.21	3.88	8979	−20.59	5.95	14,868	−8.87	2.96	4347	−13.26	3.11
F (Dry farming)	8019	−24.09	4.31	3277	−17.32	4.68	1518	−6.49	2.52	397	−13.57	3.84

**Figure 15:**
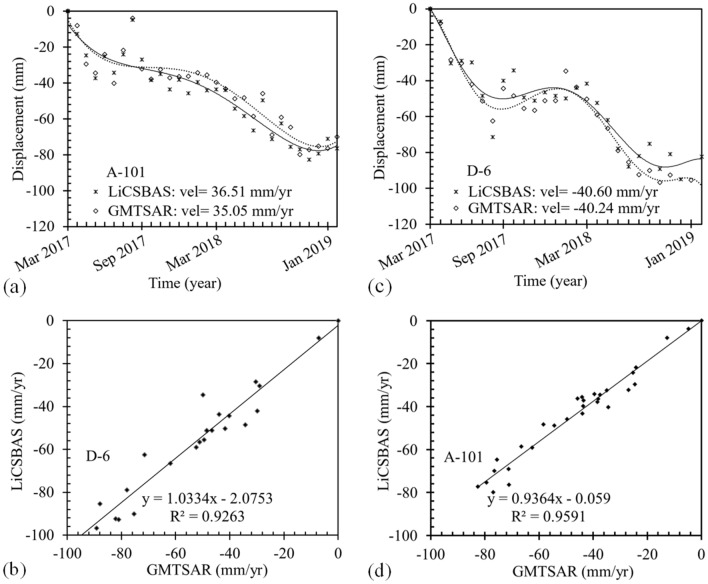
LOS displacement time series from S1A images (March 2017 to January 2019) for (**a**) A-101 track, (**b**) D-6 track, and correlation between the results of GMTSAR and LiCSBAS for (**c**) A-101 track, (**d**) D-6 track.

Figure 15 shows the annual displacement time series of an area in Class A region located in the southeast part (yellow triangle in Fig. 14) of the plain measured using LiCSBAS and GMTSAR for Tracks A-101 and D-6 for the period of March 2017 to January 2019. As presented in this figure, for the areas with expected high rates of subsidence, a good agreement is observed between the results of GMTSAR and LiCSBAS. The time series obtained from both packages indicate land subsidence with a relatively high rate of ~ 50 mm/yr. Both analysis packages show a reduction in the subsidence rate in 2019, that can be due to either implementation of some remediation measures including reduction of pumping draft and artificial recharge of aquifers from the land surface or the heavy rainfall in the region in 2019.

**Figure 16:**
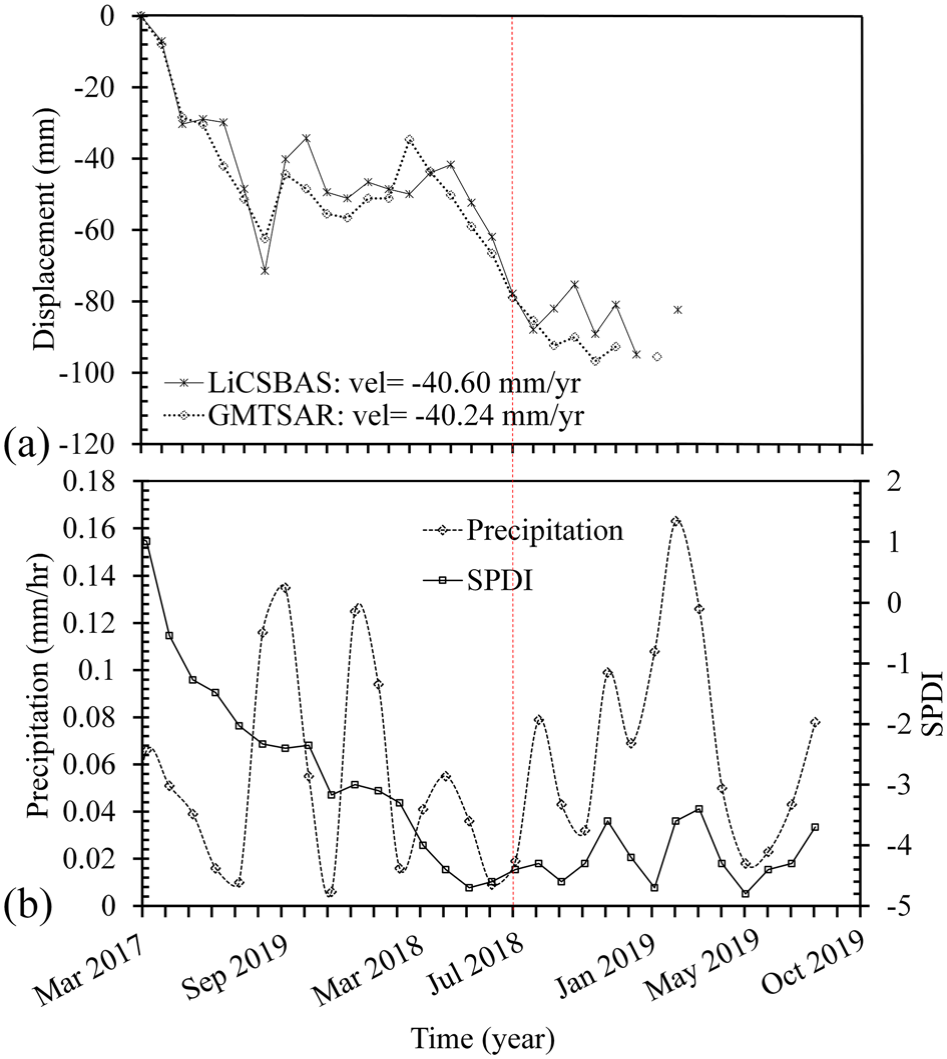
LOS displacement time series from S1A images and average annual precipitation, (severe drought from March 2017 to January 2019).

Figure 16 shows the Tropical Rainfall Measuring Mission (TRMM) and Standardized Palmer Drought Index (SPDI) of the region from 2017 to 2019. The SPDI is a monthly rainfall drought index based on calculation of the monthly cumulative and standardized rainfall anomalies^66^. SPDI is calculated using reference evapotranspiration and precipitation from the 4-km daily GRIDMET (Gridded Surface Meteorological) dataset and a static soil water holding capacity layer https://developers.google.com/earth-engine/datasets/catalog/GRIDMET_DROUGHT). Dataset presented in Fig. 16 clearly show severe drought and reduced rainfall in 2018 with TRMM and SPDI of 0.02 mm/hr and −4/8, respectively. Precipitation rate increased over the following years of 2019 and 2020, which resulted in a land subsidence decrease in the region.

**Figure 17:**
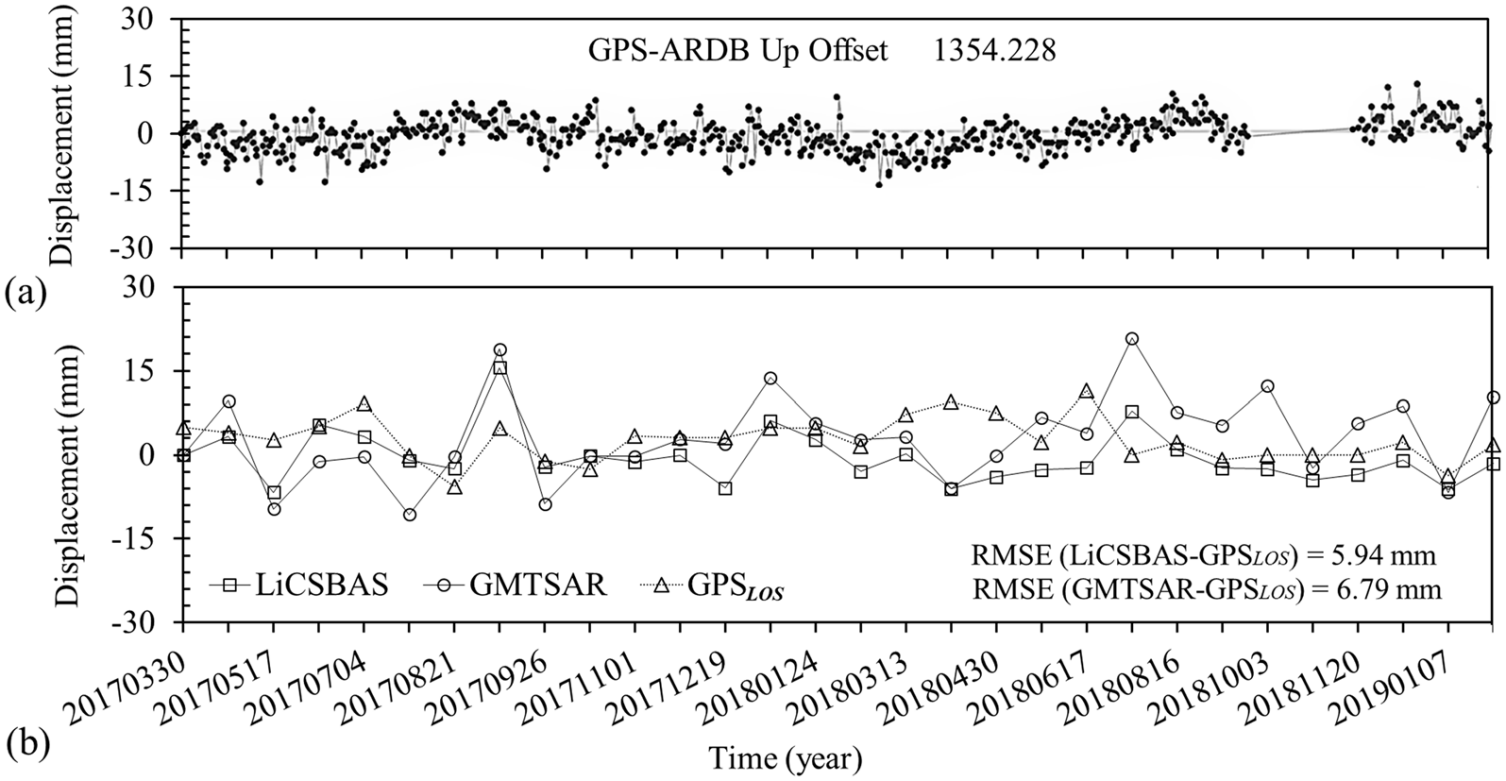
Comparison of the GPS and InSAR time-series of LiCSBAS and GMTSAR results: (**a**) Displacement trends from 2017 to 2019 of GPS permanent station (https://ipgn.ncc.gov.ir/pggn/)*.* (**b**) RMSE between the InSAR and GPS*LOS* time-series from 2017 to 2019.

In Class B region, as also presented in Table 2, differences in the displacement velocity and Std for A-101 and D-6 tracks are still small. To assess the LiCSBAS and GMTSAR results in Class B region, permanent GPS measurements were used (Fig. 17). The National Cartographic Center (NCC) of Iran (https://ncc.gov.ir), as a part of the Iranian Permanent Network for Geodynamic (IPGN) program, has established a permanent GPS station (ARDB) near Ardabil. Figure 14 shows the location of the ARDB GPS station by a red triangle. The green arrow indicates the velocity vector with an accuracy of 0.06 mm. Established in 2014, this station has been continuously measuring land deformation since then. Figure 17a shows a behavioral diagram of GPS*Up* (in vertical direction) measurements that indicates a maximum displacement rate of about 15 mm/yr from 2017 to 2019. The InSAR time-series for the location of the ARDB GPS station along with continuous GPS*LOS* (in LOS direction) measurements are illustrated in Fig. 17b. Results indicated a very similar subsidence behavior for the analyzed time series, with a root mean square error (RMSE) of around 6 mm/yr. Also, a very good agreement was observed between the InSAR and GPS*LOS* time-series fluctuations, in terms of both range and data as observed in Fig. 17b.

As presented in Table 2, the LiCSBAS displacement of Class C region for the A-101 track shows subsidence at a rate of 23 mm/yr which is in an acceptable range when compared to the 19 mm/yr subsidence rate measured using GMTSAR. In Class D and F regions, which are the areas with the lowest subsidence rates, the difference between the displacement rates estimated using the two packages were more pronounced. In these regions, displacement rate measurements estimated using LiCSBAS and GMTSAR differed about 6–7 mm/yr for the A-101 and D-6 tracks, respectively.

## Discussion

In order to evaluate the subsidence maps obtained from LiCSBAS and their compatibility with real data, it is necessary to adapt it to ground observations. In this study, relationship between land subsidence and groundwater changes, and a geotechnical-based numerical model were used to validate the results of land subsidence obtained using Envisat and S1A images by LiCSBAS.

### Relationship between land subsidence and groundwater level (GWL) changes

**Figure 18:**
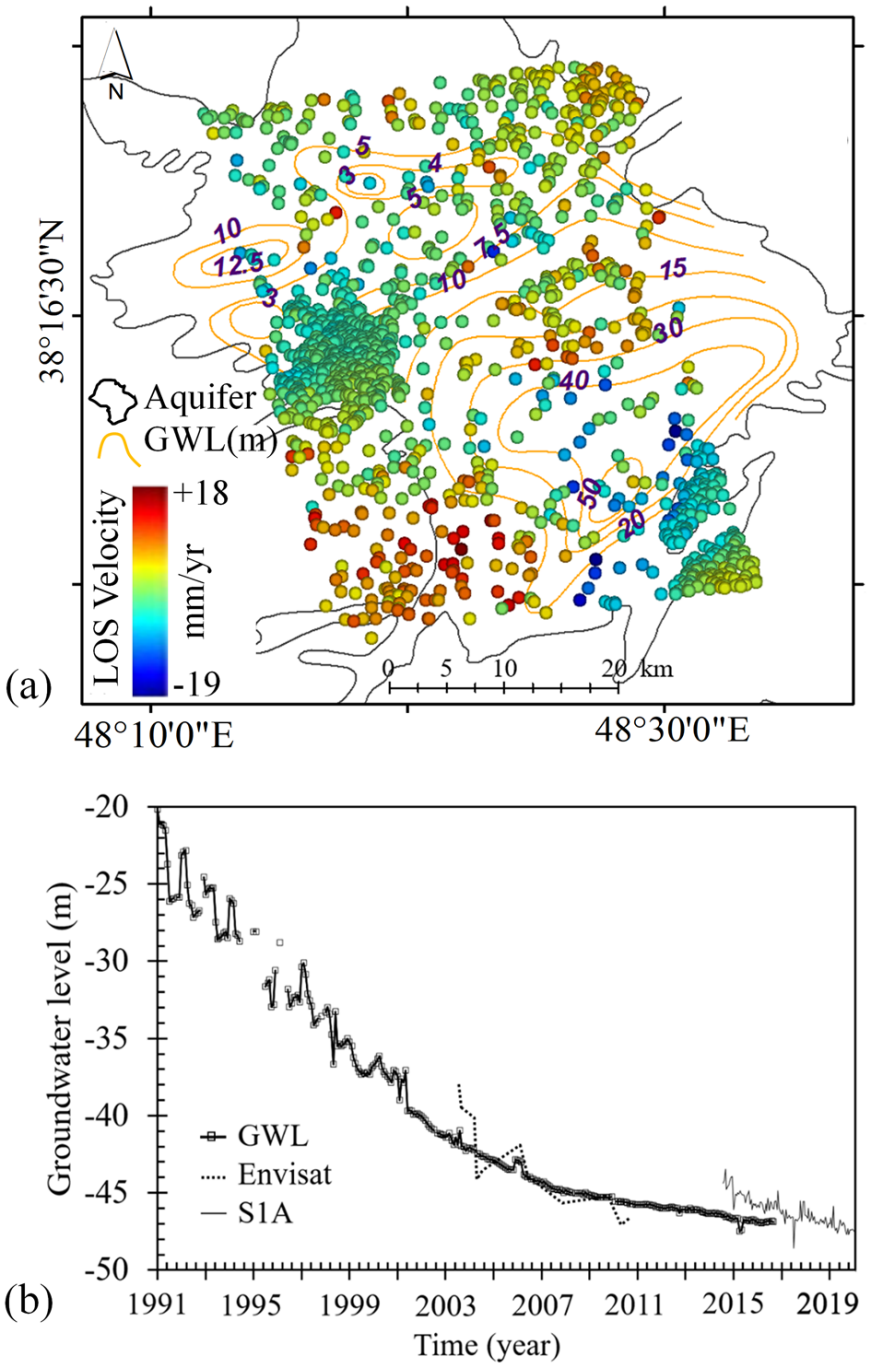
(**a**) Map for GWL changes and land subsidence of InSAR measurements. This figure was created using the QGIS version 3.14.0-Pi software (https://qgis.org/en/site/). (**b**) The trend of groundwater level changes in one of the observation wells in the Khalilabad in with the time series of subsidence.

One of the most important parameters for hydrogeological assessment of the land subsidence is the study of changes in groundwater level. Figure 18 shows contour maps of the groundwater levels and their comparison with land subsidence measurements in the region in 2006 and changes in groundwater level in Khalilabad during the years of 1991–2020. According to Fig. 18a, GWL drops from east to west of the plain, with its lowest elevation (about 50 m below the ground surface) in the southeast part of the plain. Also results indicate a 27 m drop in GWL in Khalilabad during the years of 1991–2017 (Fig. 18b). As a result of these changes in GWL along with low rates of precipitation, great rates of subsidence were recorded that particular area of the plain. According to the results of GWL change, Envisat data (2004–2010) and S1A data (2014–2020) InSAR measurements, every meter drop in GWL has resulted in about 13 mm of annual land subsidence during the years of 2003–2020. Similar observations were reported by^39,64^.

### Geotechnical numerical modeling and comparison with SBAS-InSAR results

The land subsidence measurements obtained using SBAS-InSAR results were compared with the results of a rigorous geotechnical numerical analysis of land subsidence performed using FLAC2D in Ardabil plain between the years of 2006 and 2020. FLAC2D (Fast Lagrangian Analysis of Continua in 2 Dimensions) is a numerical modeling software that implements an explicit finite volume formulation (analogous to finite-difference for 2D geometries) to capture the complex behavior of geotechnical systems and soil-structure interaction. For numerical modeling, soil layers were divided into saturated and unsaturated layers based on the location of the groundwater level and then appropriate constitutive models were assigned to each layer to properly capture the stress-deformation behavior of the layers. Terzaghi's^67^ one-dimensional consolidation theory was used for the saturated layer and the constitutive model proposed by^67^ was used for the unsaturated level. More details about^67^ constitutive model were provided in Appendix A^68^. The input parameters for the constitutive models and methods that were implemented for their measurements are presented in Table 3.

**Table 3:** The required parameters for the numerical modeling.

Parameters														
*λ*	SI*	*k*	SD*	*λs*	*ν0*	*ks*	*Cν*	*k1*	*Sr0*	*k2*	*γd*	*P'c*	Suction Stress	Groundwater Level
*λ & k*	These two parameters are obtained from the consolidation curve in which *λ* is the normalized consolidation slope in the loading mode and κ is the normal consolidation slope is in the case of loading in saturation condition of *ν*-ln *p'*													
*λs & ks*	*λs* is the initial wet and dry route slope and *ks* is the elastic section slope in Wheeler characteristic curve													
SI*&SD*	related to the air inflow (AEV) of the modified SWRC curve of the Wheeler model in two wet and dry modes													
*P'c*	known as soil pre-consolidation stress, whose value is obtained for consolidation test for different layers for soil saturation, but it should be calculated, for soils that are in the unsaturated state since the hardness or pre-consolidation stress of the soil increases by increasing the suction (decreasing the percentage of soil moisture)													
*k1 & k2*	These two parameters are used to model the interaction between mechanical and hydraulic behavior, which moves the *LC* curve when it comes to *SI* or *SD* curves, and vice versa													
*ν0 & γd*	These two parameters are measurable according to the soil Index characteristics and two parameters of porosity ratio and soil *Gs*													
*Cν*	When a time-dependent settlement estimation is required, the coefficient of consolidation should be known. The amount of this parameter can be obtained using the one-dimensional consolidation test (edometer)													

**Figure 19:**
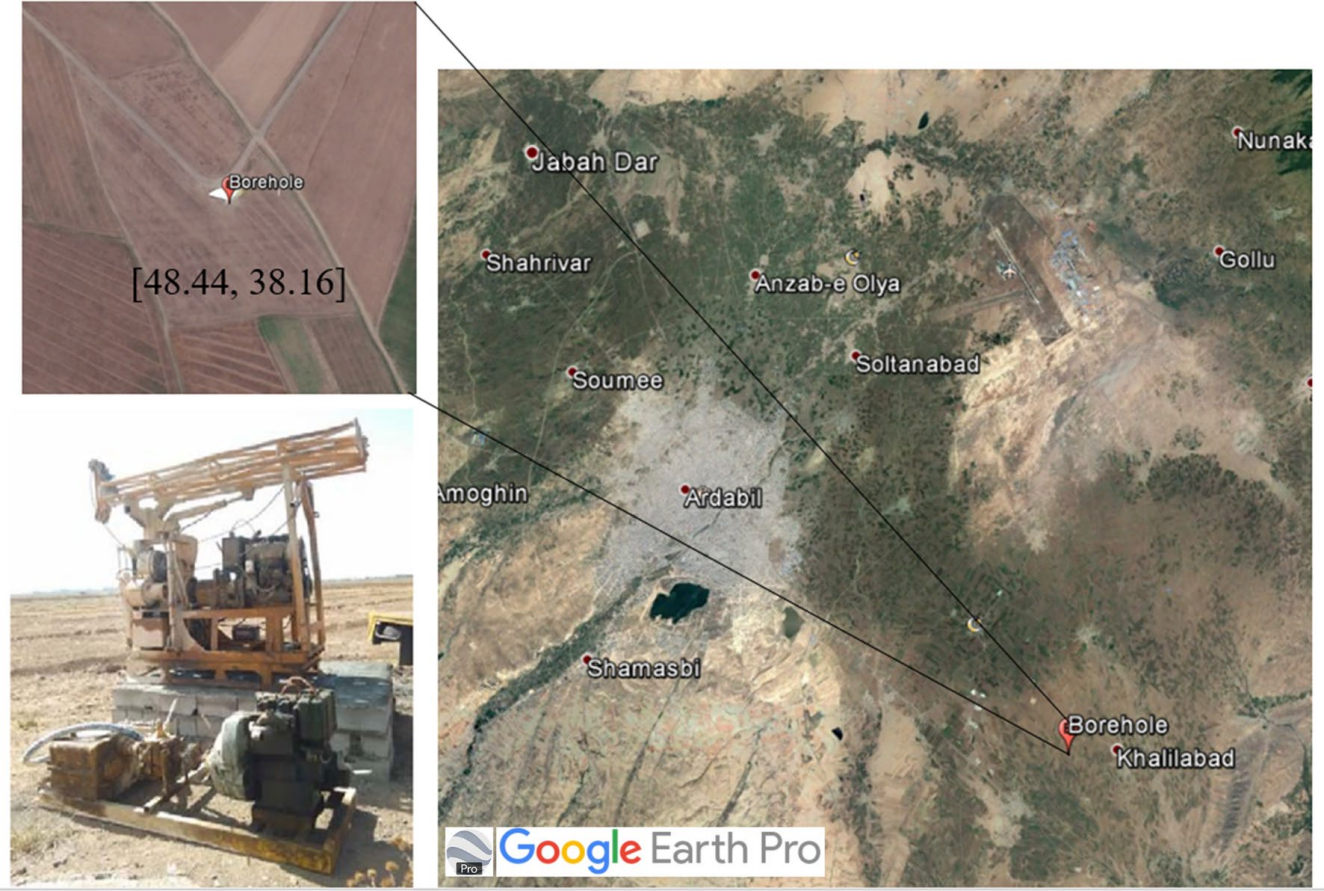
Drilling site of a borehole 105 m deep in the field. The map in this figure was created using the Google Earth/Pro version 7.3.4.8573 software (https://earth.google.com/).

As presented in Table 3, the input parameters include a series of hydraulic and mechanical properties that were mainly determined by performing a series of simple lab experiments on soil samples taken from the plain. In this study, parameters were calibrated based on properties of soil taken from a site near Khalilabad. This area has the lowest GWL and is expected to experience the highest rate of subsidence over the years. The location of the site is presented in Fig. 19. A rotatory wash-boring method was adopted to drill a 105 m borehole in the field. During boring, a SPT (standard penetration test) split sampler was used to take soil samples at 5 m depth intervals. The samples were then packed in rigid boxes and carefully transferred to the advanced soil mechanics laboratory, where index and strength tests were performed on the collected soil samples to measure input parameters for the numerical model. Five Decagon dielectric sensors were also inserted into the drilled borehole to capture and monitor profiles of soil temperature and moisture during an extended time period. The sensor measurements were then used to obtain profiles of suction and calibrate predictions of the numerical model following a procedure described by^69^. Based on the groundwater level measurements presented in Fig. 19, a groundwater level drop of 1 m/yr was assumed for the numerical analysis. Based on field measurements and sensor readings, the groundwater table was assumed to be at the depth of 50 m and a 150 m depth was considered as the depth of the bedrock. Details of soil sampling and model calibration are provided in^15^.

**Figure 20:**
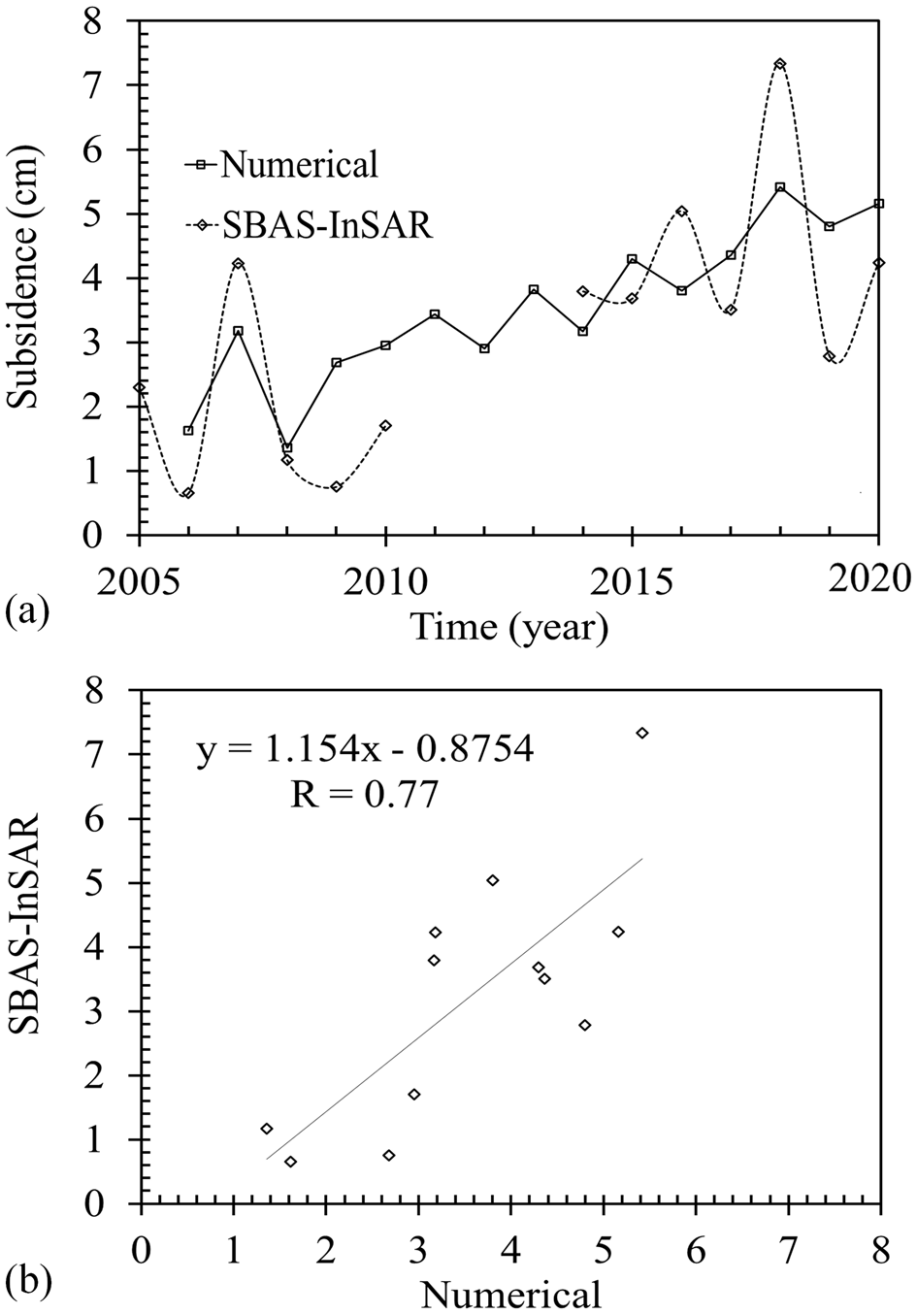
Consistency evaluation of SBAS-InSAR results: (**a**) The subsidence values of each soil layer in the Ardabil plain between 2006 to 2020. (**b**) Correlation between model predictions and InSAR measurements.

Figure 20 compares predictions of the model over the years of 2006–2020 with land subsidence measurements obtained using SBAS-InSAR results. Based on land subsidence measurements, three time periods can be considered for the study: The first period is between the years of 2006 to 2015, where, according to Fig. 20a, severe reduction in groundwater level (nearly 1.5 m/yr), resulted in considerable land subsidence in the region. From 2015 to 2018, the rate of changes in GWL decreased. However, due to long seasons of drought and significant increase in the SPDI drought index (Fig. 16b), the plain experienced significant land subsidence. Based on the results obtained using LiCSBAS, During the years of 2018 to 2020, a higher precipitation rate and some controlling measures that were taken by federal agencies decreased the rate of groundwater level drawdown to a value lower than 30 cm/yr. These changes justify lower rates of subsidence measured during the years of 2018–2020. The results of geotechnical numerical modeling showed land subsidence with an average annual rate of 3.8 cm between 2006 and 2020 which was close to what measured using SBAS-InSAR results. Results presented in this figure clearly show a good correlation between model predictions and InSAR measurements with an *R*^*2*^ value of 0.77 (Fig. 20b).

## Conclusion

In this study, an open-source interferometry time series analysis package, LiCSBAS that integrates with the automated Sentinel-1 InSAR processor was used to measure land subsidence of Ardebil plain, located at northwest, Iran, from October 2014 to January 2021. The results of this study show that LiCSBAS well performed in generating the Sentinel-1 time series over a full six years in the order of nearly 137 processed epochs for the A-101 and D-6 tracks. Using the LiCSBAS analysis package, a mean LOS velocity of 45 mm/yr was estimated for the Ardabil plain. Results also indicated subsidence rates as high as 50 mm/yr in the southeastern part of the plain. From 2018, due to higher rates of precipitation and some prevention measures, lower rates of subsidence were recorded. For consistency assessment of the land subsidence measurements using LiCSBAS, the annual displacement time series of an area near the city of Araloyebozorg (located at the southeast part of the plain) were obtained from March 2017 to January 2019 and then compared with the results of GMTSAR. LiCSBAS provided acceptable results of land subsidence in areas experiencing high rates of displacement and relatively low coherence. Comparison of the results of subsidence estimated using InSAR measurements and those obtained from GPS*LOS* measurements and a geotechnical based numerical modeling technique showed that LiCSBAS is able to accurately measure land subsidence, especially in areas that experience high rates of subsidence.

## Supplementary Information

Supplementary Information 1.

Supplementary Information 2.

Supplementary Information 3.

Supplementary Information 4.

Supplementary Information 5.

## Data Availability

All the analyzed groundwater level data, climate change and geotechnical data, and GPS observations collected during this study are included in this paper. The InSAR products that support the findings of this study include the following: and ID 006D_05111_131313 include 365 interferograms from (20141006 to 20210114): https://gws-access.jasmin.ac.uk/public/nceo_geohazards/LiCSAR_products/6/006D_05310_131313/interferograms/. LiCSAR products based on a predefined LiCSAR frame )from COMET-LiCS portal: https://comet.nerc.ac.uk/COMET-LiCS-portal/) (~ 25 GB in total): ID 101A_05193_131313 include 453 interferograms from (20141129 to 20200910): https://gws-access.jasmin.ac.uk/public/nceo_geohazards/LiCSAR_products/101/101A_05193_131313/interferograms/. Sentinel-1A data download for InSAR time series analysis by GMTSAR (from Copernicus Open Access Hub data portal: https://scihub.copernicus.eu/dhus/#/home) (~ 240 GB in total): Ascending_101 (20170330 to 20190131) and Descending_6 (20170324 to 20190113). DEM files for use with GMTSAR (SRTM DEM 30 m): https://topex.ucsd.edu/gmtsar/demgen/). The ESA website has the orbit files: (https://qc.sentinel1.eo.esa.int/aux_poeorb/). The generated datasets along with any models or codes that support the findings of this study are available from the corresponding author on reasonable request.
