# Supplementary figures and images for: Use of InSAR data for measuring land subsidence induced by groundwater withdrawal and climate change in Ardabil Plain, Iran
Source: Sci Rep. 2022 Aug 17;12:13998. doi: 10.1038/s41598-022-17438-y (PMC9385632; doi:10.1038/s41598-022-17438-y)

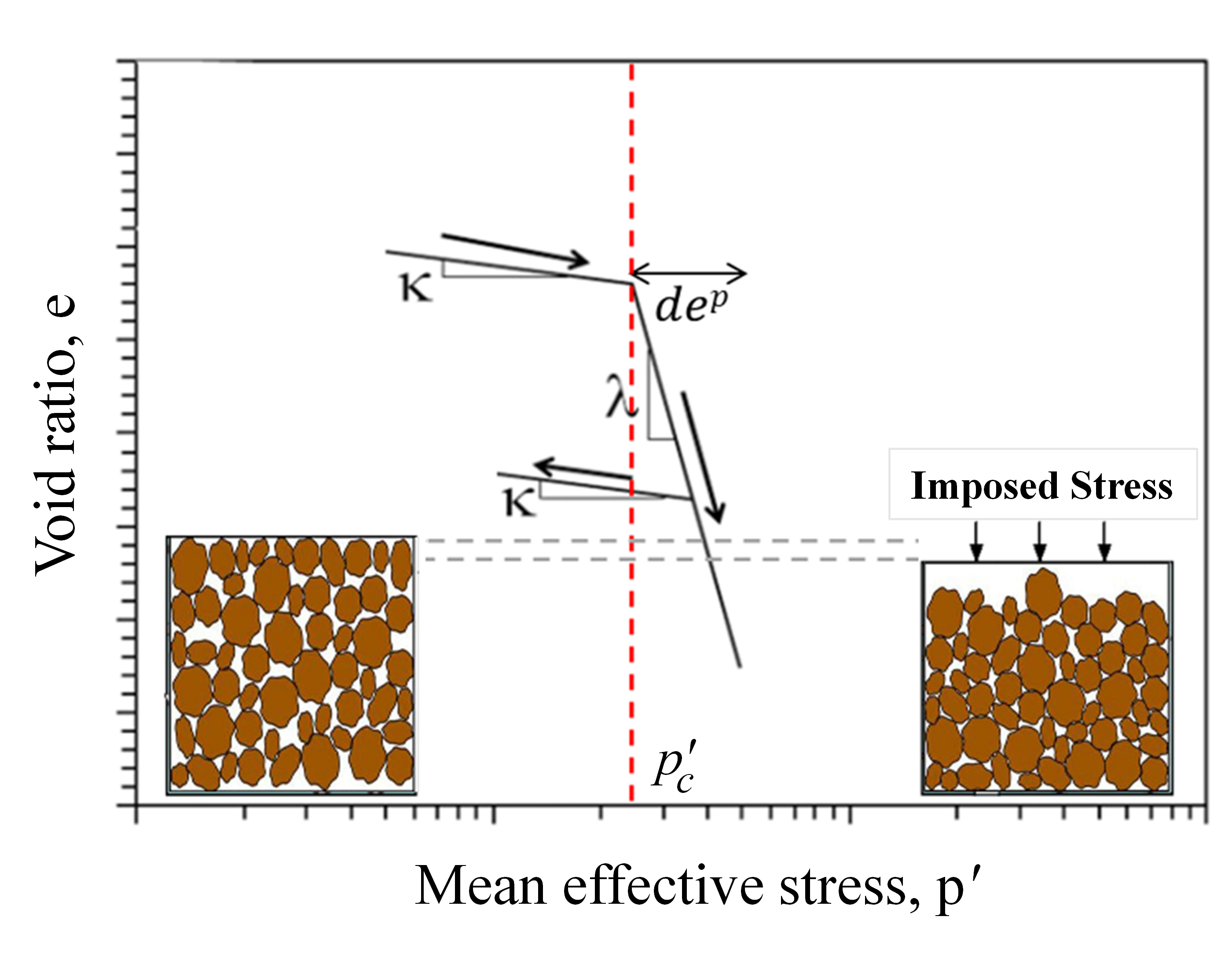

Supplement: Supplementary file 1 — Supplementary Information 1. [file 41598_2022_17438_MOESM1_ESM.tiff]

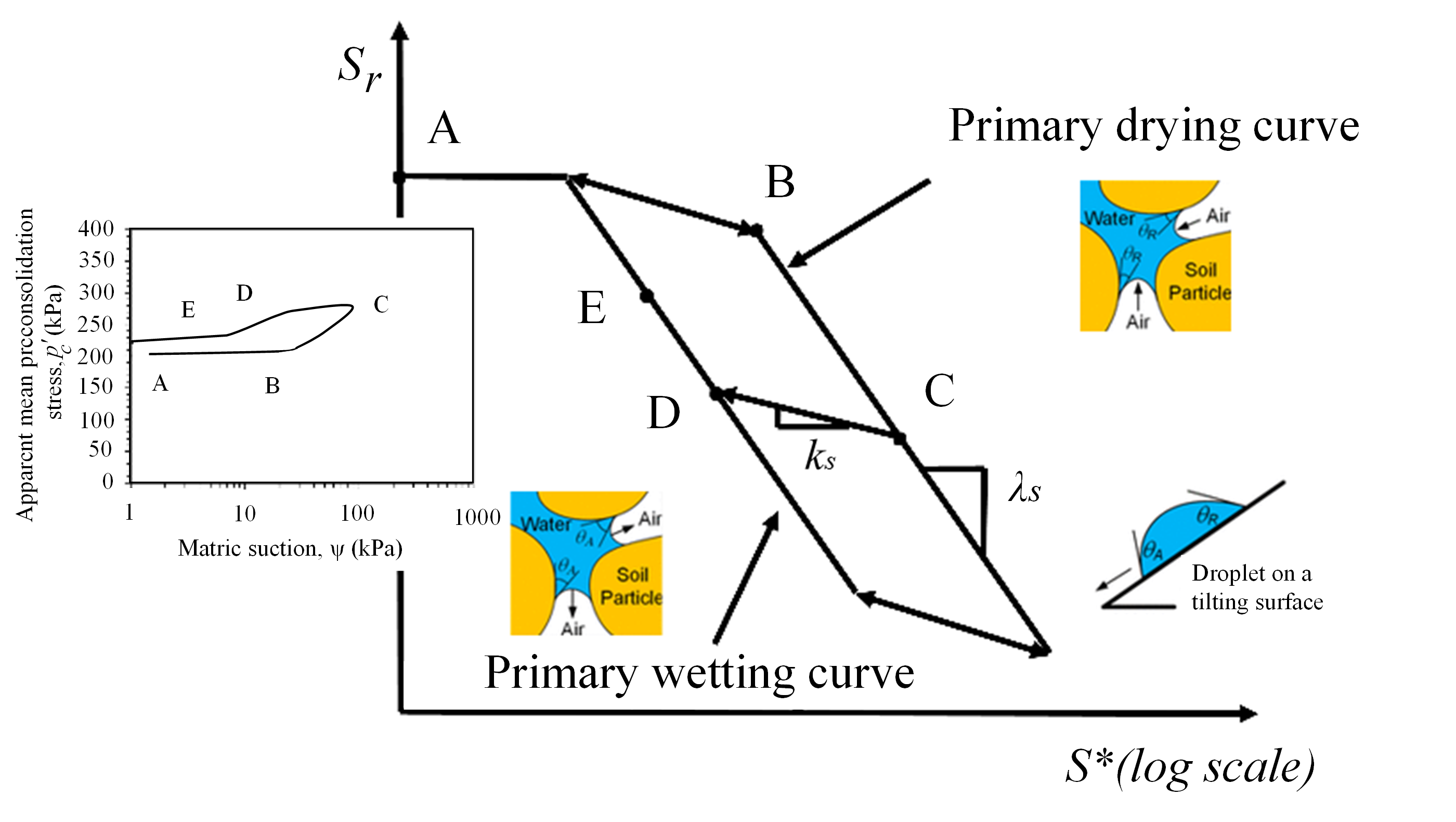

Supplement: Supplementary file 2 — Supplementary Information 2. [file 41598_2022_17438_MOESM2_ESM.tiff]

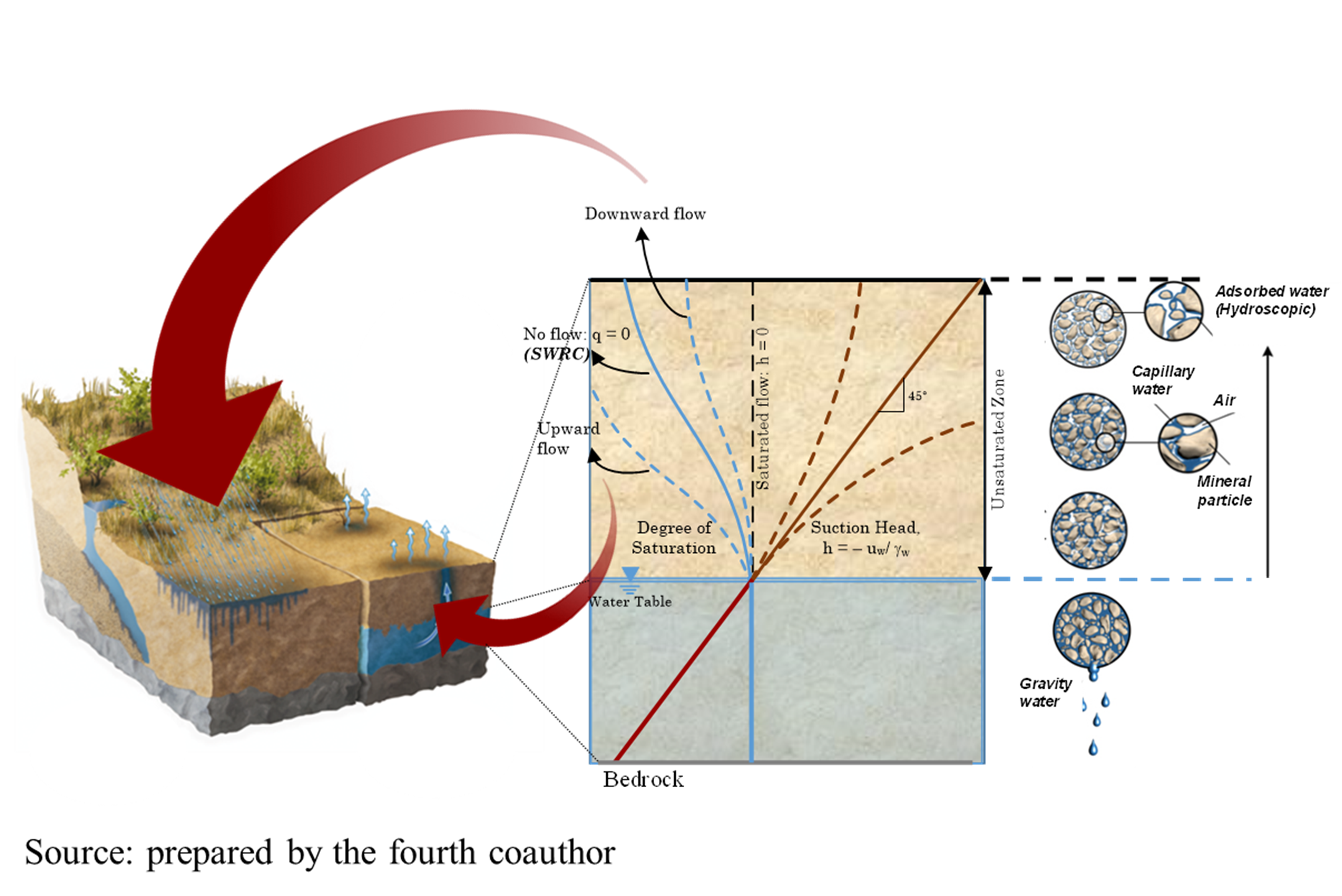

Supplement: Supplementary file 3 — Supplementary Information 3. [file 41598_2022_17438_MOESM3_ESM.tiff]
