# Supplementary material for: Use of InSAR data for measuring land subsidence induced by groundwater withdrawal and climate change in Ardabil Plain, Iran
Source: Sci Rep. 2022 Aug 17;12:13998. doi: 10.1038/s41598-022-17438-y (PMC9385632; doi:10.1038/s41598-022-17438-y)
Supplement: Supplementary file 5 — Supplementary Information 5. [file 41598_2022_17438_MOESM5_ESM.docx]

**APENDIX**

To develop an accurate modeling, soil layers are divided into saturated and unsaturated parts and an appropriate constitutive model is used for each part. Terzaghi (1943) one-dimensional consolidation theory is used for the saturated part while the constitutive model proposed by [2] is used for the unsaturated part [1].

**Governing Equations**

***Saturated Part:***

Terzaghi’s 1D consolidation theory was employed to determine soil subsidence of saturated layers. Compression of soil layers with time can be obtained from Terzaghi’s 1D consolidation equation as follows:

| $\frac{\partial u}{\partial t}=\frac{k}{m_{v}\gamma_{w}}\frac{\partial^{2}u}{\partial z^{2}}=C_{v}\frac{\partial^{2}u}{\partial z^{2}}$ | (A.1) |
| --- | --- |

in which k is the current hydraulic conductivity and C_v_ is the consolidation coefficient. Effective vertical stress variation at any elapsed time, $\Delta$σ_z_(t), is obtained according to the variation of groundwater head, $\Delta H_{T}$. Hence, the compression of soil layer, S(t), at any elapsed time, t, can be calculated as a function of the hydraulic head [3].

| $S\left(t\right)=m_{v}\Delta\sigma_{z}(t)H_{T}\approx S_{s}\Delta H(t)H_{T}$ | (A.2) |
| --- | --- |

in which H_T_ is the calculated soil layer thickness. Therefore, the compression of soil layer can be calculated with the variation of the hydraulic head from the specific storage, S_s_. The land subsidence value can be obtained by accumulating the compression of different layers of soil.

| $S_{sub}=\sum_{i=1}^{m} S(t)=\sum_{i=1}^{m} S_{si}H_{Ti}\Delta H_{i}(t)$ | (A.3) |
| --- | --- |

***Unsaturated Part:***

Problems involving groundwater flow through unsaturated soils are often analyzed using numerical models. are multiphase porous media consisting of a solid skeleton, pore water, and pore air, complicating modeling their behavior. Over the years, different approaches were developed to properly model the stress-strain behavior of unsaturated soils [2,4,5,6,7,8,9,10,11,12,]. In this study, an approach proposed by [2] was used to develop equations for the stress-strain behavior of unsaturated soils. The elasto-plastic framework by [2] is based on the concept of double hardening and coupled movements of the yield curves.

The double hardening concept describes the evolution of yield surface under the hydromechanical loading (the application of suction or mechanical load). Based on this concept, the stiffness of an infill as a geomaterial may change during two coupled physical processes: plastic displacement of the soil skeleton under applied mechanical load (Figure A.1), and the hydraulic process of water flow during wetting and drying.

**Figure A.1.** Elasto-plastic changes in volume due to compression

In soils undergoing any of these processes, a new arrangement of particles with different values of void ratio and degree of saturation are expected, and consequently, the soil skeleton may experience a more/less stabilized state against yielding under subsequent loading and unloading. The movement of the yield surface under the hydromechanical loading can be represented using the expression described by [13] as follows:

|  | (A.4) |
| --- | --- |

where and are the values of over-consolidation stress at current and saturated conditions, respectively, $\Delta e^{p}$ is a plastic change in void ratio, λ is the slope of the virgin compression curve, and κ is the slope of the elastic rebound curve, S_e_ is the effective saturation, and b is referred to as the double-hardening parameter which governs the rate of change in caused by changes in S_e_.

Soil water chracteristic curves (SWCCs) that relate matric suction (pore air pressure minus pore water pressure) and water content demonstrate hysteretic behavior. Hysteresis in the SWCC refers to the non-unique relationship between the soil’s matric suction and its water content, whereby the soil can have two different water contents at the same matric suction value, depending on the preceding sequence of wetting and drying. Such hysteresis is thought to be caused by various factors, such as variable and irregular cross-sections of the pores, the different contact angles in the advancing and receding soil-air interface menisci, and the difference in entrapped air volume at different matric suction values [14]. Figure A.2 shows different processes experienced by unsaturated soil and in Figure A.3, possible effects of hydraulic hysteresis on the over-consolidation ratio has been presented. Furthermore, the stress-strain behavior of unsaturated soils is elastoplastic. Wheeler et al. (2003) proposed an elasto-plastic framework to model the water retention curve’s hysteresis. In this study, the elasto-plastic model proposed by [2] has been incorporated in a fully coupled hydro-mechanical FD model for unsaturated soils developed by the authors. The framework of the elasto-plastic model is presented briefly in the following paragraph, and is followed by its implementation in the FD model.

**Figure A.2.** Different processes experienced by unsaturated soils

**Figure A.3.** Variation in soil’s degree of saturation and stiffness during hydraulic hysteresis [13]

***Stress and Strain Variables***

The first stress variable of this framework is expressed as:

| $\sigma_{ij}^{*}=\sigma_{ij}-\left. \left[ S_{r}u_{1}-\left( 1-S_{r} \right)u_{a} \right] \right.\delta_{ij}$ | (A.5) |
| --- | --- |

| $\sigma_{ij}^{*}=\sigma_{ij}-\left[S_{r}u_{1}+\left(1-S_{r}\right)u_{a}\right]\delta_{ij}$ | (A.6) |
| --- | --- |

in which, *σij* is the total stress tensor and *σ***ij* is the stress tensor usually defined as Bishop's stress [15]. In addition to *σ***ij*, the modified suction (s* = ns) is applied as the second stress variable for the effect of the meniscus water [16]. Therefore, the porosity (n) is incorporated into the stress state variables rather than with the strains. The model applies the following stress state variables:

| $p^{*}=p-S_{r}u_{1}-\left( 1-S_{r} \right)u_{a}$ | Mean Bishop Stress | (A.7) |
| --- | --- | --- |
| $s^{*}=ns$ | Modified Suction | (A.8) |
| $q$ | Deviator stress in the case of anisotropic loading | (A.9) |

The advantage of the stress variables is that they give more power to model the behavior of soils as s^*^ includes porosity (n) and *P*^*^ includes the degree of saturation (S_r_). Another advantage of using Bishop's stress is retrieving to the saturated effective stress when the soil changes to saturated conditions even if the suction is not zero.

***Mathematical Formulation of the Constitutive Model***

The elasto-plastic framework by [2] is based on the coupled movements of the yield curves. As shown in Figure A.4, yielding on LC curve causes plastic volumetric strain, followed by an upward movement of the SI and SD curves. In addition, a plastic decrement of S_r_ is occurred followed by an upward movement of the SD curve and outward movement of the LC curve. Yielding to SD causes a plastic increment of $S_{r}$ followed by coupled downward movement of the SI curve and inward movement of the LC curve. Yielding on either SI or SD is not associated with plastic volumetric strains [17].

**Figure A.4.** Yield surfaces during the hydromechanical loading considered by [2]

As shown in Figure A.4, yielding on LC curve causes plastic volumetric strain, followed by an upward movement of the SI and SD curves. In addition, a plastic decrement of S_r_ is occurred followed by an upward movement of the SD curve and outward movement of the LC curve. Yielding to SD causes a plastic increment of $S_{r}$ followed by coupled downward movement of the SI curve and inward movement of the LC curve. Yielding on either SI or SD is not associated with plastic volumetric strains [17]. The elastic and plastic volumetric strains are defined as:

| $d\varepsilon_{\nu}^{e}=\frac{\kappa dp^{*}}{\nu p^{*}}$ | (A.10) |
| --- | --- |
| $d\varepsilon_{\nu}^{e}=\frac{\left( \lambda-\kappa\right)dp_{0}^{*}}{\nu p_{0}^{*}}$ | (A.11) |

In which, $\kappa$is the slope of unloading—reloading line and $\lambda$ is the slope of virgin consolidation line in the in p': *v* space. The flow rule for the LC curve is expressed as:

| $\frac{dS_{l}^{p}}{d\varepsilon_{l}^{p}}=0$ | (A.12) |
| --- | --- |

The elastic (reversible) and plastic (irreversible) increments of the saturation degree are given by:

| $\frac{dS_{r}^{e}}{d\varepsilon_{l}^{p}}=-\frac{\kappa_{s}ds^{*}}{s^{*}}$ | (A.13) |
| --- | --- |
| $dS_{r}^{p}=-\left( \lambda_{s}-\kappa_{s} \right)\frac{ds_{r}^{*}}{s_{r}^{*}}=-\left( \lambda_{s}-\kappa_{s} \right)\frac{ds_{D}^{*}}{s_{D}^{*}}$ | (A.14) |

Accordingly, $\kappa_{s}$ is an elastic constant regarding the changes in the modified suction $s^{*}$ and $\lambda_{s}$ is the slope of the virgin consolidation line in the *lns** space. A typical water retention curve is shown in Figure A.3. The two primary curves for drying and wetting have a slope of $\lambda_{s}$ and the elastic lines between them have a slope of $\kappa_{s}$.

The flow rule for the SI and SD curves is explained as:

| $\frac{d\varepsilon_{p}^{p}}{dS_{r}^{p}}=0$ | (A.15) |
| --- | --- |

The coupled movements of LC, SI, and SD curves are governed by two coupling factors k_1_ and k_2_. The corresponding movements of SI and SD curves in the case of soil yields on LC curve are given by:

| $\frac{dp_{0}^{*}}{p_{0}^{*}}=k_{1}\frac{ds_{r}^{*}}{S_{r}^{*}}=k_{1}\frac{ds_{D}^{*}}{S_{D}^{*}}$ | (A.16) |
| --- | --- |

The corresponding movements of LC curve in the case of yields on S_r_ or S_D_ curve are given by:

| $\frac{ds_{r}^{*}}{s_{r}^{*}}=\frac{ds_{D}^{*}}{S_{D}^{*}}=k_{2}\frac{dp_{0}^{*}}{p_{0}^{*}}$ | (A.17) |
| --- | --- |

According to Equations A.11, A.14 and A.16 the movement of the LC curve is the sum of any direct yielding on the LC curve and the coupled movements due to the movements of SI or SD curve:

| $\frac{dp_{0}^{*}}{p_{0}^{*}}=\frac{\nu d\varepsilon_{p}^{p}}{\left( \lambda-\kappa\right)}=\frac{k_{1}dS_{r}^{p}}{\left( \lambda_{s}-\kappa_{s} \right)}$ | (A.18) |
| --- | --- |

In the same way, the movement of the SI or SD curve is the sum of any direct movements due to yielding on SI or SD curve and the coupled movements due to movements of LC curve:

| $\frac{dS_{D}^{*}}{S_{D}^{*}}=\frac{dS_{r}^{*}}{S_{r}^{*}}=\frac{k_{2}\nu d\varepsilon_{p}^{p}}{\left( \lambda-\kappa\right)}+\frac{dS_{r}^{p}}{\left( \lambda_{s}-\kappa_{s} \right)}$ | (A.19) |
| --- | --- |

***References***

1. Terzaghi K. *Theory of consolidation. Theor Soil Mech.* 265–296 (1943).
2. Wheeler, S. J., Sharma, R. S., & Buisson, M. S. R. Coupling of hydraulic hysteresis and stress–strain behaviour in unsaturated soils. *Géotechnique*. 53(1), 41-54 (2003).
3. Shen, S. L., Xu, Y. S., & Hong, Z. S. Estimation of land subsidence based on groundwater flow model. *Marine Georesources and Geotechnology*. 24(2), 149-167 (2006).
4. Alonso, E. E., Gens, A., & Josa, A. A constitutive model for partially saturated soils. *Géotechnique*. 40(3), 405-430 (1990).
5. Chávez, C., & Alonso, E. E. A constitutive model for crushed granular aggregates which includes suction effects. *Soils and foundations*. 43(4), 215-227 (2003).
6. Roy, S., & Rajesh, S. Simplified model to predict features of soil–water retention curve accounting for stress state conditions. *International Journal of Geomechanics*. 20(3), 04019191 (2020).
7. Sheng, D., Sloan, S. W. & Gens, A. A constitutive model for unsaturated soils: thermomechanical and computational aspects. *Computational Mechanics*. 33(6), 453-465 (2004).
8. Sun, D. A., Sheng, D. & Sloan, S. W. Elastoplastic modelling of hydraulic and stress–strain behaviour of unsaturated soils. *Mechanics of Materials*. 39(3), 212-221 (2007).
9. Vanapalli, S. K., Pufahl, D. E. & Fredlund, D. G. Interpretation of the shear strength of unsaturated soils in undrained loading conditions. *In Proceedings of the 52nd Canadian Geotechnical Conference, Regina, Sask* (pp. 25-27) (1999, October).
10. Wan, R. G. & Guo, P. J. A simple constitutive model for granular soils: modified stress-dilatancy approach. Computers and Geotechnics, 22(2), 109-133 (1998).
11. Wheeler, S. J. & Karube, D. Constitutive modelling. *In proceedings of the first international conference on unsaturated soils/unsat'95/paris/france/6-8. volume 3* (1996).
12. Xiong, Y. L., Ye, G. L., Xie, Y., Ye, B., Zhang, S. & Zhang, F. A unified constitutive model for unsaturated soil under monotonic and cyclic loading. *Acta Geotechnica*. 14(2), 313-328 (2019).
13. Khosravi, A. & McCartney, J. S. Impact of hydraulic hysteresis on the small-strain shear modulus of low plasticity soils. *Journal of Geotechnical and Geoenvironmental Engineering.* 138(11), 1326-1333 (2012).
14. Bashir, R., Sharma, J., & Stefaniak, H. Effect of hysteresis of soil-water characteristic curves on infiltration under different climatic conditions. *Canadian Geotechnical Journal*. 53(2), 273-284 (2015).
15. Bolzon, G., Schrefler, B. A. & Zienkiewicz, O. C. Elastoplastic soil constitutive laws generalized to partially saturated states. Géotechnique, 46(2), 279-289 (1996).
16. Houlsby, G. T. The work input to an unsaturated granular material. *Géotechnique*. 47(1), 193-196 (1997).
17. Javadi, A. A. & Elkassas, A. S. I. Numerical modeling of hydraulic hysteresis in unsaturated soils*. Transport in porous media*. 85(2), 521-540 (2010).
